# Development and validation of a new measurement instrument to assess internship experience of medical doctors in low-income and middle-income countries

**DOI:** 10.1136/bmjgh-2023-013399

**Published:** 2023-11-08

**Authors:** Yingxi Zhao, Sulaiman Jalloh, Phung Khanh Lam, Yakubu Kevin Kwarshak, Daniel Mbuthia, Nadine Misago, Mesulame Namedre, Nguyễn Thị Bé Phương, Sefanaia Qaloewa, Richard Summers, Kun Tang, Raymond Tweheyo, Bridget Wills, Fang Zhang, Catia Nicodemo, David Gathara, Mike English

**Affiliations:** 1 NDM Centre for Global Health Research, Nuffield Department of Medicine, University of Oxford, Oxford, UK; 2 Ola During Children's Hospital, Freetown, Sierra Leone; 3 Oxford University Clinical Research Unit, Ho Chi Minh City, Viet Nam; 4 University of Medicine and Pharmacy at Ho Chi Minh City, Ho Chi Minh City, Vietnam; 5 Department of Surgery, Division of Urology, Jos University Teaching Hospital, Jos, Plateau State, Nigeria; 6 KEMRI-Wellcome Trust Research Programme, Nairobi, Kenya; 7 Interdisciplinary Research Group in Public Health / Doctoral School, University of Burundi, Bujumbura, Burundi; 8 Independent Researcher, Suva, Fiji; 9 College of Medicine, Nursing and Health Sciences, Fiji National University, Suva, Fiji; 10 School of Social Policy, University of Birmingham, Birmingham, UK; 11 Vanke School of Public Health, Tsinghua University, Beijing, People's Republic of China; 12 Department of Health Policy Planning and Management, Makerere University School of Public Health, Kampala, Uganda; 13 Centre for Health Systems Research and Development (CHSRD), The University of Free State, Bloemfontein, South Africa; 14 Department of Endocrinology and Metabolism, Peking University People’s Hospital, Beijing, People's Republic of China; 15 Nuffield Department of Primary Care Health Sciences, University of Oxford, Oxford, UK; 16 Department of Economics, Verona University, Verona, Italy; 17 MARCH Centre, London School of Hygiene and Tropical Medicine, London, UK

**Keywords:** Medical education, internship experience, scale development, measurement, low- and middle-income countries

## Abstract

Routine surveys are used to understand the training quality and experiences of junior doctors but there are lack of tools designed to evaluate the training experiences of interns in low-income and middle-income countries (LMICs) where working conditions and resource constraints are challenging. We describe our process developing and validating a ‘medical internship experience scale’ to address this gap, work involving nine LMICs that varied in geographical locations, income-level and internship training models. We used a scoping review of existing tools, content validity discussions with target populations and an expert panel, back-and-forth translations into four language versions and cognitive interviews to develop and test the tool. Using data collected from 1646 interns and junior medical doctors, we assessed factor structure and assessed its reliability and validity. Fifty items about experiences of medical internship were retained from an initial pool of 102 items. These 50 items represent 6 major factors (constructs): (1) clinical learning and supervision, (2) patient safety, (3) job satisfaction, (4) stress and burnout, (5) mental well-being, and (6) fairness and discrimination. We reflect on the process of multicountry scale development and highlight some considerations for others who may use our scale, using preliminary analyses of the 1646 responses to illustrate that the tool may produce useful data to identify priorities for action. We suggest this tool could enable LMICs to assess key metrics regarding intern straining and initial work experiences and possibly allow comparison across countries and over time, to inform better internship planning and management.

**Summary box**

- Internship experience can be challenging due to the rapid transition from medical school to clinical practice, especially long working hours, high workloads and constant new learning and assessment.
- Countries like the UK and US conduct routine surveys of their doctors in training, led by regulators, to understand their experiences and monitor and report on training quality. However, most low-income and middle-income countries (LMICs) do not have similar routine surveys and there is a relative lack of research on internship experiences in these countries.
- With collaborators from 9 LMICs, we developed a 50-item Medical Internship Experience Scale (MIES) based on data from 1646 medical interns and junior doctors from LMICs.
- MIES is reliable and valid and broadly covers six major constructs, that is, clinical learning and supervision, patient safety, job satisfaction, stress and burnout, mental wellbeing, fairness and discrimination, and this tool could be used by governments, medical schools and regulators to compare internship experiences across different training facilities and to identify specific areas where improvements are needed.

## Introduction

Medical internship is the period when doctors in training transition from medical education into clinical practice typically before they become licensed and registered as independent medical practitioners. The programme of internship may differ across countries: in the UK, Modernising Medical Careers was introduced in 2005 to create a 2-year Foundation Programme^1^; in many low-income and middle-income countries (LMICs), internship is a 1-year stand-alone programme leading to licensure^2^; while for countries like the US, internship usually refers to the first year of residency where qualified doctors undertake graduate medical education to obtain a license for a chosen specialty.^3^

Despite the different terminology in each setting, interns often work long hours while learning and being assessed.^3^ Interns also need to shift their identity to that of physicians and take on new responsibilities and challenges. Some may lack confidence in their ability to manage uncertainty when responsible for others’ lives especially when facing sudden patient deaths.^4–7^ Such factors can result in rapid burnout, stress and other mental health problems.^4 8 9^ In LMICs due to resource constraints, interns may also experience low availability of essential medicines and equipment, limited supervision and feedback,^10^ poor safety climate, extremely poor working conditions and work without pay.^11^ For example, Erasmus described interns in South Africa as ‘slaves of the state’.^12^

Countries like the UK and US conduct routine surveys of their doctors to understand their training experiences and its quality, led by the General Medical Council^13^ and Accreditation Council for Graduate Medical Education,^14^ respectively. These surveys are relatively broad, for example, spanning perspectives on teamwork, workload and patient safety. More recently in 2018, the UK General Medical Council added a burnout inventory to its survey.^13^

In comparison, most LMICs do not have such routine surveys, confirmed by a scoping review of quantitative tools that measure internship experiences.^15^ Only 14 out of 92 included studies were conducted in LMICs. The review also revealed lack of common definitions of key areas to measure, and substantial variation in the questions in major national trainee surveys,^13 14 16^ limiting options for comparison across countries. Existing tools from high-income settings might not capture significant differences in context in LMICs: for example, poor infrastructure and material resources availability in internship hospitals^17^ are common in LMICs. Therefore, we aimed to develop and validate a tool, the ‘medical internship experience scale (MIES)’, focusing on the internship experience of medical doctors in LMICs.

## Development of the MIES

We followed Boateng’s 9-step scale development and validation framework for the development of MIES.^18^ Collaborators from nine countries were involved in different stages of scale development and validation processes (table 1). Eight of them contributed data to a final survey sample alongside some responses from non-study countries. The nine study countries varied in geographical locations, income-level and internship training models, which allowed us to understand and develop a tool suited for use across settings. An overview of the scale development and validation process is provided in table 2, with step-by-step detail provided in online supplemental appendix 1. Information on the nine study countries is provided in table 1.

10.1136/bmjgh-2023-013399.supp1 Supplementary data

**Table 1:** Overview of the nine study countries for MIES development and validation process

Country	Kenya	Uganda	Burundi	Sierra Leone	Nigeria	South Africa	Fiji	China	Vietnam
Income level (World Bank 2021)	Lower middle income	Low income	Low income	Low income	Lower middle income	Upper middle income	Upper middle income	Upper middle income	Lower middle income
Language version	English	English	French	English	English	English	English	Mandarin Chinese	Vietnamese
Site	Nationwide with various sizes of hospitals	Kampala, Lira with various sizes of hospitals	Bujumbura and Kibuye with major large hospitals	Freetown, Makeni and Bo with major large hospitals	Jos with various sizes of hospitals	N/A	Suva, Labsa, Lautoka with various sizes of hospitals	Nationwide with various sizes of hospitals	Ho Chi Minh City with major large hospitals
Local ethics approval	Kenya Medical Research Institute (KEMRI) (SERU 4071)	Makerere University School of Public Health Research and Ethics Committee (SPH-2021-168)	University of Burundi (05/05/2021)	Sierra Leone Ethics and Scientific Review Committee	Jos University Teaching Hospital in Nigeria (JUTH/DOS/REC/127/2693)	N/A	Fiji National University	Tsinghua University (No. 20220063)	University of Medicine and Pharmacy at Ho Chi Minh City (No. 708)
Survey mode	Online/REDCap	Online/REDCap	Paper	Paper	Online/Microsoft Forms	N/A	Online/Microsoft Forms	Online/Wenjuanxing	Online/Microsoft Forms
Participation in intern content validity discussion	X	X	X	X		X		X	X
Participation in cognitive interview	X	X	X (French)	X		X		X (Chinese)	X (Vietnamese)
Participation in survey	X	X	X	X	X	N/A	X	X	X
Sample size	358	487	120	96	90	N/A	42	160	177
% Male	51%	69%	56%	67%	49%	N/A	38%	35%	56%
% current intern	37%	13%	93%	57%	24%	N/A	76%	70%	54%
Mean age (SD)	28.4 (2.3)	28.0 (2.8)	27.9 (2.5)	32.1 (4.8)	28.9 (3.2)	N/A	27.5 (2.5)	23.9 (1.4)	26.6 (1.5)

”N/A” indicates that South Africa-specific survey was not conducted. Additionally, an open survey was conducted to supplement the scale development process in non-study countries and a total of 113 samples were included in the final sample (see online supplemental appendix for the non-study countries source).

MIES, Medical Internship Experience Scale.

**Table 2:** Overview of MIES development and validation process

Step	Detail
Phase 1: Item development	
Step 1: Identification of Domain and Item Generation: Selecting Which Items to Ask	Primarily a deductive approach, domain identified and items generated from a scoping review on existing tools that measure medical internship experience (Patient Health Questionnaire-9,^19^ Perceived Stress Scale,^20^ Professional Quality of Life,^21^ Postgraduate Hospital Educational Environment Measure^22^ and Safety Attitude Questionnaire^23^) with additional questions focusing on challenges most common to LMICs such as physical resources and patient safety 102 items all standardised to 5-point Likert scale, either ‘very often – often – sometimes – rarely - never’ or ‘strongly agree – agree – neutral – disagree – strongly disagree’
Step 2: Content Validity: Assessing if the Items Adequately Measure the Domain of Interest	Evaluation by target population: discussion with a total of 43 medical interns in 7 countries to understand whether the domains and items represent the actual experience from interns. Items revised based on interns’ feedback Evaluation by experts: discussion with experts who were either clinicians with responsibility for training/supervision of interns, and/or researchers who have familiarity with survey/scale development processes (n=14) to rate item for content relevance, representativeness, technical quality from 1 (not relevant), 2 (low relevance/needs major revision), 3 (medium relevance/needs minor alteration) to 4 (high relevance). Item-level content validity index (I-CVI) were calculated and 17 items with a below 78% I-CVI dropped or revised.
Phase 2: Scale development	
Step 3: Pretesting Questions: Ensuring the Questions and Answers Are Meaningful	Translation into three additional languages (Mandarin Chinese, Vietnamese and French) Cognitive interviews with 19 medical interns in 7 countries for face validity and ensure that the respondents understand items as we intended. Interviews were conducted using a mix of ‘think aloud’ (tell me what you are thinking as you answer this question) and ‘probing’ (what this term X means to you and why you chose that answer) and items were further revised and rephrased at this phase.
Step 4: Survey Administration and Sample Size: Gathering Enough Data from the Right People	Survey self-administered online (using REDCap or Microsoft forms) or paper, with sample collected from eight study countries including Kenya, Uganda, Burundi, Nigeria, Sierra Leone, Fiji, Vietnam and China as well as a global survey Study population is the current cohort of medical interns or junior medical officers who finished internships in 2018 or after, identified using a mix of snowballing and purposive sampling approach A total of 1646 complete responses were collected as of January 2023, with additional 77 responses dropped due to missing 10% of scale items
Step 5: Item Reduction: Ensuring the Scale Is Parsimonious	No item had an over 10% missing rate for the overall sample, missing data replaced by median due to data skewess and low frequency Classical test theory (CTT) to select items based on interitem correlations. Using a cut-off of 0.3, 6 items were further dropped as they have very low correlations
Step 6: Extraction of Factors: Exploring the Number of Latent Constructs that Fit Your Observed Data	Kaiser-Meyer-Olkin (KMO) measure of sampling adequacy (high 0.97) and Bartlett’s test significant for fitness of factor analysis Exploratory factor analysis conducted on remaining factors, with six factors explaining 90% of variance. 32 items were removed due to cross-loading, leaving a final 50-item scale
Phase 3: Scale evaluation	
Step 7: Tests of Dimensionality: Testing if Latent Constructs Are as Hypothesised	Confirmatory factor analysis on the sample showed CFI (0.89) is less than satisfactory while RMSEA (0.05) and SRMR (0.05) are satisfactory Multigroup confirmatory factor analysis for measurement equivalence suggested that model fitted poor for configural invariance. Therefore, there is difference in terms of factor loading across different countries. This could be due to various reasons including adequate but still small sample size, cross-loading of items, etc
Step 8: Tests of Reliability: Establishing if Responses Are Consistent When Repeated	Cronbach’s alpha is a measure of internal consistency; how closely items are related within a group. For the overall final scale items was 0.95 (excellent) and ranged from 0.74 (acceptable) to 0.93 (excellent) for the six identified factors. The scale-specific and factor-specific alpha results were similar for most countries.
Step 9: Tests of Validity: Ensuring You Measure the Latent Dimension You Intended	We examined the validity of MIES in line with Cook’s recommendation Content validity of MIES was ensured as the item development process included a scoping review to identify relevant tools, as well as content validity discussions with both the target population and an expert panel Response process refers to how well the respondents’ response aligns with the intended construct, and we used cognitive interviews as part of pilot testing to ensure that respondents understand the items as we designed Evidence on the internal structure of the scale derived from internal consistency and factor structure analysis We do not yet have evidence on relations to other variables and consequences for MIES scale, but the tool could be used in the future to identify hospitals that trained interns with poorer internship experience and further improve internship training environment

CFI, comparative fit index; RMSEA, root mean square error of approximation; SRMR, standard root mean square residual.

### Item development

First, we conducted a scoping review of the tools that measure medical internship experience that is reported elsewhere.^15^ We summarised the major themes examined by 92 studies and identified three domains of interest: well-being, educational environment and work conditions and environment. We generated an item pool through reviewing existing tools and indicators from the scoping review, adapting five commonly used tools (table 2). We supplemented these with additional questions on physical resources and patient safety. We standardised this initial set of items (n=102) and responses intending that they measure the broad internship experience and are collectively compatible.

We assessed content validity with the target population and experts.^18^ For target population, we conducted discussions with 43 interns in 7 countries to understand whether the domains and items were relevant to internship experiences. Items were revised, added or dropped at this stage (online supplemental appendix 2). We then conducted validation involving 14 purposefully selected experts on medical training and/or scale development to evaluate items for content relevance, representativeness and technical quality (online supplemental appendix 3). We retained 88 items after this phase (online supplemental appendix 4).

### Scale development

The tool was translated into three additional languages (Mandarin Chinese, Vietnamese, and French, online supplemental appendix 4), led by study country collaborators using forward and back translation.^19^ We then conducted pretesting and cognitive interviews^20 21^ with 19 medical interns to ensure that the items are meaningful to the target population and that the MIES survey could be successfully administered. Pretesting was conducted using country collaborators’ proposed mode (online or paper), in English or translated language and alongside cognitive interviews. Items were further revised and rephrased at this phase.

We then moved onto the survey administration stage. The analytical sample was collected from eight study countries (excluding South Africa) using a mix of snowballing and purposive sampling approaches as well as through an open survey shared via social media and colleagues (table 1). The study population eligible for the MIES survey was the cohort of medical interns or junior doctors in 2022 who finished internships in 2018 or after. The survey was self-administered by participants either online or using paper-based questionnaires. A total of 1646 complete responses were collected and used as our analytical sample, out of which 113 samples were collected from 14 non-study countries through the open survey. Overall, the mean age of the study sample was 27.8 years. Thirty-nine per cent of the sample were interns at the time of survey administration (on average having completed 7 months internship) and the rest were within 3 years post internship. We acknowledge that our survey sample includes some interns yet to complete their internship and doctors up to 3 years post internship that could influence our findings although further analysis did not suggest any obvious differences linked to time post internship (online supplemental appendix 9). Table 2 presents the characteristics of the sample by country, notably countries like Burundi, China and Fiji had a higher proportion of current interns, and only 13% of Ugandan respondents were current interns as one cohort just recently completed internship at the time of survey.

For selecting the items, no items had an over 10% missing rate for the overall sample; therefore, we kept all items and replaced the missing values with the median of each item. We then conducted item reduction analysis using inter-item and item-total correlations as techniques in line with classical test theory. Six items were dropped because of very low correlations with other items, potentially because they were not measuring similar constructs or not fully understood by participants. Details on each of the items tested in the analytical sample could be found in online supplemental appendix 5.

We used factor analysis to understand the latent structure of the items.^18^ After confirming the Kaiser-Meyer-Olkin and Bartlett’s test for the fitness of data for factor analysis, we conducted exploratory factor analysis (EFA) and used scree plots and the variance explained by the factor model and the factor loading pattern to determine the number of factors to retain.^18 22^ Six factors had Eigen values exceeding one, an inspection of the Scree plot however, revealed a likely break after the third factor. We decided to retain the six-factor solution because it comprised fewer items overall (50 vs 65) and in our opinion had a more intuitive domain structure. Furthermore, the three-factor-solution did not significantly outperform the six-factor solution (online supplemental appendix 6 and 7). We further removed 32 items with cross-loading across three rounds of testing.

Based on these results, and after reading the items, we named the six factors: (1) clinical learning and supervision; (2) patient safety; (3) stress and burnout; (4) job satisfaction; (5) mental well-being; and (6) fairness and discrimination, respectively. Final items and their corresponding factor loading as well as sources are presented in table 3.

**Table 3:** Final items included for the MIES scale

Factor	Question	Item loading	Adapted or new
Factor 1—clinical learning and supervision (n=14) (Cronbach’s alpha: 0.93)	My clinical supervisors are enthusiastic about teaching and supervision.	0.93	PHEEM
	The clinical supervisors provide me with regular feedback.	0.90	
	My clinical supervisors are accessible for teaching and supervision.	0.89	
	My clinical supervisors have good mentoring skills.	0.88	
	The clinical supervisors provide me with feedback on my strengths and weaknesses to ensure my professional development.	0.75	
	My clinical supervisors have good communication skills.	0.74	
	I have enough clinical learning opportunities for my needs during the internship period.	0.68	
	I have good clinical supervision at all times during my internship.	0.67	
	My clinical supervisors encourage me to be an independent learner.	0.63	
	My clinical supervisors have set clear expectations.	0.61	
	I am able to participate actively in educational sessions (e.g., continuing medical educations) during my internship.	0.59	
	I have opportunities to acquire the appropriate practical procedures for clinical practice during my internship.	0.58	
	I have access to educational sessions and programmes that are relevant to my needs during my internship	0.58	
	My clinical supervisors promote an atmosphere of mutual respect.	0.55	
Factor 2—patient safety (n=10) (Cronbach’s alpha: 0.90)	There are clear and updated patient safety protocols in the internship hospital.	0.81	New
	Medical errors are handled appropriately in my internship hospital.	0.73	SAQ
	I know the proper channels to direct questions regarding patient safety.	0.72	
	I am encouraged by my colleagues to report any patient safety concerns I may have.	0.70	
	The culture in my internship hospital makes it easy to learn from the errors of others.	0.69	
	I would feel safe being treated as a patient in my internship hospital.	0.68	
	I know the proper channels to direct questions regarding my own safety.	0.65	New
	There are adequate infection prevention and control measures.	0.63	New
	I can report any concern and receive responsive feedback in my internship hospital.	0.63	New
	There are clear clinical protocols and guidelines across all departments in the internship hospital.	0.46	PHEEM
Factor 3—stress and burnout (n=10) (Cronbach’s alpha: 0.90)	I feel overwhelmed because my case workload seems endless during the internship.	0.97	ProQOL
	I feel worn out because of my work as a medical intern.	0.91	
	I feel trapped by my job as a medical intern.	0.71	
	I feel bogged down and held back by the internship hospital.	0.68	
	I am preoccupied by concerns about multiple patients during my internship.	0.67	
	I find it difficult to separate my personal life from my life as a medical intern.	0.63	
	I feel that I am unable to balance my work and personal life during my internship.	0.63	PSS
	I feel nervous and/or stressed because of my internship work.	0.63	
	I find that I could not cope with all the work that I had to do during my internship.	0.45	
	I am angered because of things that were outside of my control.	0.40	
Factor 4—job satisfaction (n=7) (Cronbach’s alpha: 0.88)	I am proud of what I can do to help as a medical intern.	0.93	ProQOL
	I believe I can make a difference through my work.	0.88	
	I believe that I am a success as a medical intern.	0.81	
	I am happy that I chose to do this work.	0.79	
	My internship work makes me feel satisfied.	0.73	
	My ability to keep up with clinical techniques and protocols makes me feel pleased.	0.64	
	My internship experience met my expectation.	0.48	New
Factor 5—mental well-being (n=5) (Cronbach’s alpha: 0.84)	I have eating problems, either have poor appetite, or have been overeating.	0.82	PHQ-9
	I have sleeping problems, either have trouble falling or staying asleep, or sleeping too much.	0.76	
	I have trouble concentrating on things either work-related, or outside of my work.	0.66	
	I have little interest or pleasure in doing things that I used to enjoy.	0.63	
	I feel down, depressed, or hopeless because of my internship work.	0.59	
Factor 6—fairness and discrimination (n=4) (Cronbach’s alpha: 0.74)	There is gender discrimination in my internship hospital.	0.73	PHEEM
	There are other forms of discrimination (e.g., ethnicity, religion, tribe, disability) in my internship hospital.	0.73	New
	I get bullied or victimised within my internship hospital.	0.47	New
	I have to perform inappropriate tasks during my internship.	0.44	PHEEM

PHEEM stands for Postgraduate Hospital Educational Environment Measure, ProQOL stands for Professional Quality of Life Measure, PHQ9 stands for Patient Health Questionnaire 9, PSS stands for Perceived Stress Scale and SAQ stands for Safety Attitude Questionnaire. Items are either adapted from these scales or are new.

### Scale evaluation

We then moved on to examining dimensionality. We primarily focused on measurement invariance, that is, whether the psychometric properties are generalisable across different subgroups—in our case across different countries. We first ran the confirmatory factor analysis on the overall sample (n=1646) again and in each individual country. The overall sample comparative fit index (CFI, 0.89) was less than satisfactory while root mean square error of approximation (0.05) and standard root mean square residual (SRMR, 0.05) were satisfactory. The results were similar for the 4 countries with over 150 respondents (online supplemental appendix 1).We then conducted multigroup confirmatory factor analysis in countries with over 150 respondents’ (Kenya, Uganda, Vietnam and China sample, n=1182). Due to the less-than-ideal CFI in the overall sample, model fit was moderate for configural invariance (CFI=0.86). This suggests some differences in terms of factor loading across different countries. This could be due to relatively small sample sizes, cross-loading of items, or other reasons. Comparing results such as aggregate scores across different countries therefore requires extra caution as the scales might perform slightly differently in each country.

For reliability, we calculated Cronbach’s alpha to assess the internal consistency of the scale items, which is one commonly used reliability criterion. Cronbach’s alpha for the overall final scale items was 0.95 and ranged from 0.74 to 0.93 for the six identified factors (table 3). The scale-specific and factor-specific alpha results were similar for most countries. We examined the validity of MIES in line with Cook’s recommendation^23^ (table 2).

## Lessons and implications for practice

In summary, we developed and validated a scale to measure the internship experience of medical doctors in LMICs. We tested for reliability, factor structure and validity across four languages and different countries varying in internship training contexts. Fifty items that comprise the scale broadly cover six major constructs, that is, clinical learning and supervision, patient safety, stress and burnout, job satisfaction, mental well-being, and fairness and discrimination. The scale developed is built on several existing tools such as Postgraduate Hospital Educational Environment Measure (PHEEM; 40 items covering 3 constructs of autonomy, teaching and social support),^24^ Perceived Stress Scale (PSS; 10 items with 1 construct)^25^ and Professional Quality of Life Measure (ProQOL; 30 items covering 3 constructs of compassion satisfaction, burnout and secondary traumatic stress).^26^ However, these existing tools include items not specific to internship training and/or not relevant to LMICs.^15^ We therefore reviewed and revised items through content validity discussions with junior doctors in LMICs, an expert panel and cognitive interviews. Table 3 provides the information on whether the final items are adapted from existing tools or developed as new, for example, items under clinical supervision and learning are all adapted from PHEEM, items under stress and burnout are all adapted from PSS and ProQOL whereas nearly half of the patient safety items are newly added.

### Reflection on the multicountry scale development process

Scale development is an iterative, complicated and onerous process, and often requires researchers to make their own decisions in terms of what methods and approach to use. Our scale development was conducted in a multi-country and multi-language setting, which itself presented additional analytical and logistics challenges.

At the item development stage, we conducted rounds of target population content validity discussions and cognitive interviews as well as an expert panel to ensure cross-cultural equivalence of the items. The cognitive interviews were especially helpful in ensuring face and content validity to revise or remove redundant, ambiguous or difficult-to-understand items.^20^ However, this resulted in a few items being dropped at this stage because they were not universally relevant. This included items on pay and remuneration as some countries do not pay their interns since internship is considered part of preservice education.

We faced logistics issues when surveying across countries as this required use of different data collection platforms (paper-based, REDCap, Microsoft Forms, Wenjuanxing) and languages as well as different considerations in survey advertisement, recruitment and incentives. For example, in Uganda, data were collected online supplemented by field visits to 4 major hospitals in a 3-month period and resulted in a substantial sample size (n=487). In Kenya, we partnered with the professional association, regulator and major medical schools, and advertised through their channels offering 1 GB mobile data as an incentive. Despite a lengthy process over 8–9 months aiming to reach all eligible respondents, we estimate that we received complete responses from approximately 15% of the eligible population. We hoped for a minimum of 150 responses from each country to support invariance analysis. However, Sierra Leone has roughly 50 interns per year and so achieving our proposed sample size would be extremely challenging.

### Examples of using the MIES

**Figure 1:**
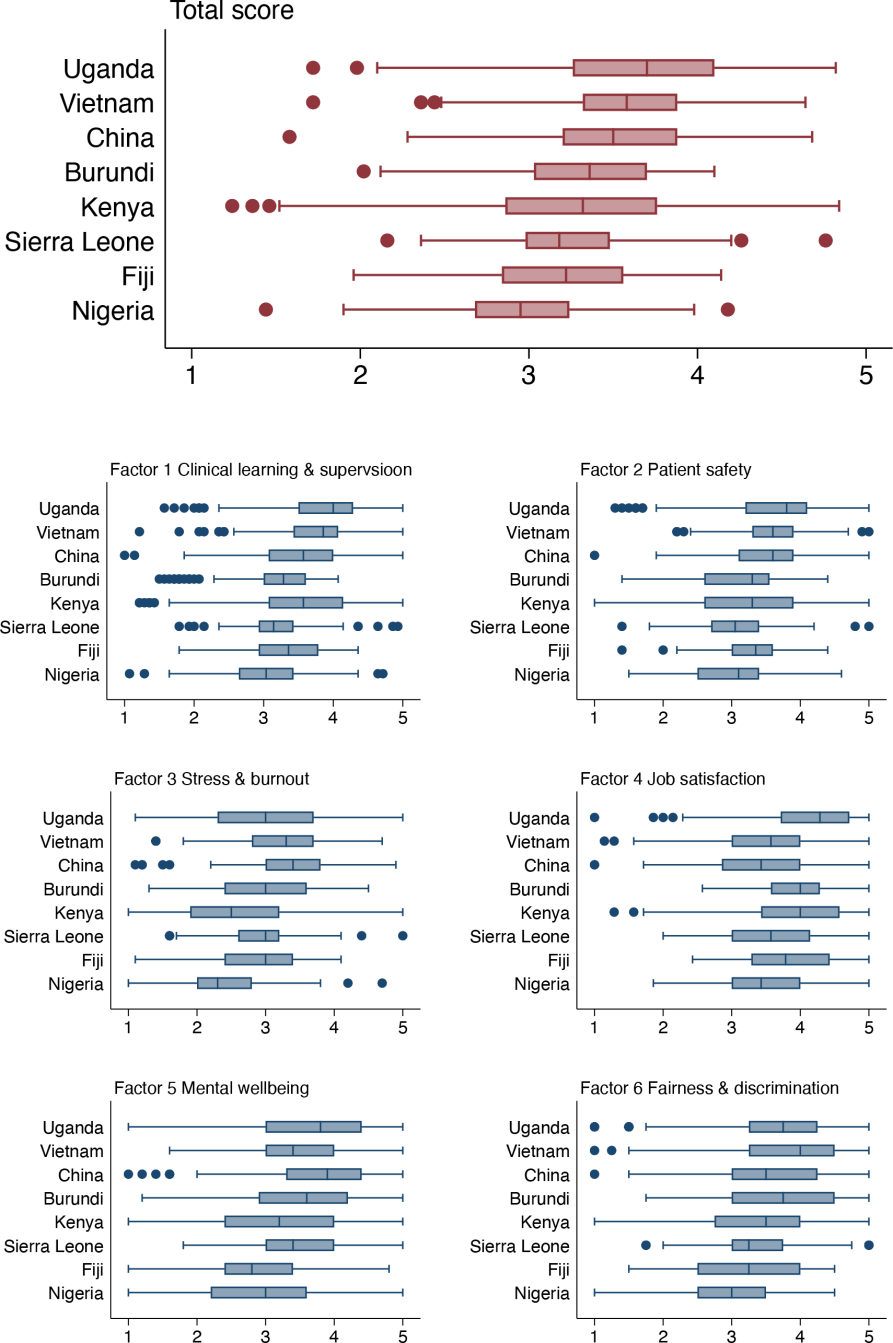
Aggregate score by factors in selected countries. All ‘negative’ items have been reversed and higher score in each factor is more favourable.

Our newly developed tool could enable LMICs to assess key human resource metrics for interns and possibly allow comparison across countries. As shown in figure 1 by calculating and comparing the aggregate scores of the six factors (after reversing negatively scored items), we observed a difference in the total scores (all with a maximum score of 5) between the eight study countries. Job satisfaction (factor 4) was perhaps surprisingly rated relatively high across all countries (range from median 3.43 in Nigeria and China to 4.29 in Uganda) whereas stress and burnout (Factor 3) might be considered concerning (range from median 2.30 in Nigeria to 3.40 in China). While these data suggest differences between countries, further testing of the psychometric properties of this tool and improved representativeness of sampling would be important to confirm findings. Further illustration of the potential value of MIES is shown in table 4. Ranking those items scored lowest and highest from the global and country-specific samples suggests, other than China and Vietnam, that stress and well-being are of concern with interns unable to balance their work and personal life and constantly stressed. Such results indicate that improving interns well-being and relieving workload and stress is an urgent agenda in several countries.^27 28^ Interestingly, interns were generally proud of their work, believing they could make a difference. However, responses from China and Vietnam may suggest that efforts are needed to increase junior doctors’ job satisfaction and sense of personal accomplishment. The low job satisfaction might be attributed to broader issues such as tense doctor–patient relationship and threatened professional identity as seen in China.^29–33^

**Table 4:** Questions/items that were ranked lowest and highest based on responses to all six factors and using ‘global’ data from all countries

		Global	Uganda	Kenya	Vietnam	China	Burundi	Sierra Leone	Nigeria	Fiji
The 5 Items with lowest ranked scores using data from all countries	I feel that I am unable to balance my work and personal life during my internship.	**1**	1	1	3	15	16	5	1	2
	I feel nervous and/or stressed because of my internship work.	**2**	5	2	1	3	13	14	2	1
	I am preoccupied by concerns about multiple patients during my internship.	**3**	2	5	5	14	1	1	4	10
	I feel worn out because of my work as a medical intern.	**4**	4	3	9	4	4	11	3	4
	I feel overwhelmed because my case workload seems endless during the internship.	**5**	3	4	16	28	19	3	7	9
The 5 Items with highest ranked scores using data from all countries	I am proud of what I can do to help as a medical intern.	**1**	2	1	24	44	1	2	1	3
	I believe I can make a difference through my work.	**2**	1	2	34	34	2	1	4	4
	My ability to keep up with clinical techniques and protocols makes me feel pleased.	**3**	3	3	33	4	6	15	2	2
	I believe that I am a success as a medical intern.	**4**	4	4	40	30	10	5	3	10
	I have opportunities to acquire the appropriate practical procedures for clinical practice during my internship.	**5**	8	5	8	16	25	14	6	6

The five lowest ranked items (1=lowest of all to 5=fifth lowest, in red) are presented with the five highest ranked items (1=highest of all to 5=fifth highest, in green) with respect to all 50 items. In the country specific columns, the rank is given for that item with respect to responses to all 50 items within that country.

As many countries face workforce challenges, we suggest that governments, medical schools or licensing bodies could use the MIES tool to assess internship experiences in a country, potentially across different training facilities to identify areas where improvements are needed. For example, our preliminary data suggest significant differences in interns’ experience of patient safety and job satisfaction between facilities of different sizes in Kenya (online supplemental appendix 8). The tool could also be used to track changes in internship experiences over time, perhaps administered as an annual internship exit survey, or to explore cross-country differences. It also has the possibility of being linked with other datasets such as workforce registries, similar to the annual national training survey and UK medical education database, to inform workforce planning and support education and training regulation.^34^

### Additional considerations for others using the scale

We opted for a six-factor structure for MIES instead of three factor. Both versions have their pros and cons as they performed similarly in subsequent dimensionality and reliability testing and both had a slightly poor model fit that is common in studies running confirmatory and EFA in the same sample.^35^ The six-factor version has 50 items, is shorter despite having more domains and therefore easier to implement. We also felt its domain labels were intuitive, perhaps helping prompt appropriate actions. One advantage of the three-factor version might be that it includes items related to resource availability. In some settings, such items may be important to add, for example, ‘the internship hospital has adequate supply of diagnostics, equipment and medication for my study and work needs’ (Q88) and ‘the internship hospital has good quality accommodation for me when on call’ (Q85). We provide further information on the 3 and 6 factor domains and items, and the original 88 items (online supplemental appendix 6). Countries designing internship surveys could add in further questions of specific interest such as those on remuneration and career intentions as stand-alone issues.

One key limitation is the measurement equivalence of the scale across different countries. According to the confirmatory factor analysis and the multigroup confirmatory factor analysis, configural invariance is less than ideal suggesting that the factor number and loading pattern could be slightly different across countries. This could be due to various reasons including relatively small sample sizes and cross-loading of items. While we conducted content validity discussions and cognitive interviews in different countries to ensure items are similarly understood, some items might have been interpreted differently in different countries. Second, we acknowledge that different data collection approaches in our study countries could lead to potential biases, for example, two countries used a paper-based survey and some countries only sampled participants from major hospital sites due to logistics considerations. Third, despite reducing the item number to 50, the tool is still relatively long and would require 15–30 min to answer, which could be a disadvantage for busy interns leading to survey dropout. We also acknowledge that despite having nine countries with varied geographical locations, income-level, and internship training models in the scale development process, there could be other context-specific factors influencing the internship experiences in other countries. Future studies should explore other types of validity and reliability of this tool including test–retest reliability, psychometrics in other countries and perhaps conduct further testing to produce a shorter form of the tool.

## Conclusion

In conclusion, we developed and validated a scale to measure the internship experience of medical doctors in LMICs. We tested for its reliability, factor structure and validity across four languages and eight different countries varying in internship training contexts. The final six-factor scale includes 50 items that broadly cover six major constructs, that is, clinical learning and supervision, patient safety, job satisfaction, stress and burnout, mental well-being, fairness and discrimination. This tool could be used to inform better internship planning and management especially improving junior doctors’ experiences during internship.

## Data Availability

Data are available upon reasonable request. The datasets generated during and/or analysed during the current study are available from the corresponding author on reasonable request.
