# Supplementary material for: Development and validation of a new measurement instrument to assess internship experience of medical doctors in low-income and middle-income countries
Source: BMJ Glob Health. 2023 Nov 8;8(11):e013399. doi: 10.1136/bmjgh-2023-013399 (PMC10632816; doi:10.1136/bmjgh-2023-013399)
Supplement: Supplementary data [file bmjgh-2023-013399supp001.pdf]

Supplementary appendix 1. Step-by-step information of the MIES scale development and validation process

Step 1: Identification of Domains and Item Generation: Selecting Which Items to Ask

The first step of item development is to identify the domains and the items to be included in the scale. Through the scoping review of the tools that measure medical internship experience, we identified three major domains of interest: well-being, educational environment, and work condition and environment.

For item generation, our item pool was identified using a deductive approach, i.e., reviewing existing tools and indicators from the scoping review. Five tools were selected (PHQ-9, PSS, ProQOL, PHEEM and SAQ) and permissions to use these tools were secured from relevant individuals and organisations. We also added additional questions that we believe are of relevance, with support for these from early findings of our research – for example, additional questions on physical resources and patient safety. A total of 102 items were included for MIES, with 3 additional screening questions and 4 socio-demographic questions. We provisionally categorised them into several sub-domains that might be stand-alone factors. Table 1 provides an overview of the domains, subdomains, item generation pool and the number of items.

We also standardised the questions and responses to ensure that they measure the broad internship experience and are compatible within a survey. First, we changed the introductory paragraph and items to ask medical interns and junior doctors to reflect on their whole internship period not specific to any specialty rotation, instead of “over the past two weeks” or “past month” such as in the PHQ-9 (1) and PSS (2). Second, some of the original scales have a four-point Likert scale and some others a five-point scale. To allow better response patterns and easier comparison, all the items were standardised into a five-point Likert scale, either “very often – often – sometimes – rarely - never” or “strongly agree – agree – neutral – disagree – strongly disagree”.

Table 1. Item generation, existing tools and number of items

Domain	Sub-domain	Item generation pool	Number of items
**Well-being**	Stress	Perceived stress scale	10
	Depression	Patient health questionnaire 9	9
	Burnout	Professional quality of life (ProQOL)	30
	Fatigue	ProQOL	-
	Satisfaction	ProQOL	-
**Educational environment**	Teaching and supervision	Postgraduate Hospital Educational Environment Measure (PHEEM)	40
	Organisational support	PHEEM	-

**Work condition and environment**	Work condition	PHEEM	-
	Physical resource	PHEEM + additional questions	2
	Safety culture	Safety Attitude Questionnaire (SAQ, safety domain only)	10
		**Total scale items**	**102**
**Non-scale items**			
**Screening (start)**			3
**Socio-demographic (end)**			4

Step 2: Content Validity: Assessing if the Items Adequately Measure the Domain of Interest

Content validity refers to "the adequacy with which a measure assesses the domain of interest" (3) and specifies that items are relevant and representative (4). We followed Boateng et al.'s recommendation and assessed content validity through evaluation by the target population and by experts (4).

For target population evaluation, we conducted discussions in 7 countries with 5-12 medical interns each, a total of 43 interns, to understand whether the domains and items represented the actual internship experiences they had. These discussions were conducted online in English (except for China which is in a mix of Chinese and English) where we went through all the proposed items, asked them if they were relevant and probed for any other potential items or ideas. Items were revised and/or dropped at this stage leading to another version with 101 items (see Supplementary appendix 2. Changes to MIES items from original tools to post-content validity discussion).

For expert panel validation, we conducted a joint expert-panel discussion involving 14 experts to evaluate items for content relevance, representativeness and technical quality. Experts were either clinicians with responsibility for training/supervision of interns, and/or researchers who have familiarity with survey/scale development processes. These experts were selected purposefully, either the country local collaborators themselves if they were not involved in the previous steps, or recommended by collaborators, and came from 10 countries (see Supplementary appendix 3. Expert panel members involved in scale evaluation). We conducted the expert panel discussion in English where we first introduced the MIES project and asked experts to rate all 101 items based on "whether this item is most relevant to capture the internship experience of the medical doctors, across the broad domains of wellbeing, educational environment, work condition & environment" from 1 (not relevant), 2 (low relevance / needs major revision), 3 (medium relevance / needs minor alteration) to 4 (high relevance); Ratings were collected online using Microsoft Forms and there was a free-text column after each section where participants could provide comments and suggest revision of items.

We calculated two content validity indices: the item-level content validity index (I-CVI), which is the proportion of experts giving items a relevance of 3 or 4; and scale-level content validity index (S-CVI) based on the average method, which is the average of all the I-CVI scores for all items; and compared our results with the recommended values from Yusoff (5) and Lynn (6). The S-CVI was 86% which was satisfactory and above the 78% cut-off recommendation by Yusoff (5) and Lynn (6) considering we have more than 9 experts. 17 items had a below 78% I-CVI, of which 13 were dropped and 4 were revised. We also conducted some follow-up discussion with the group after the rating exericise and based on the discussion, we re-worded “clinical teacher” to “clinical supervisor”; and also added “during my internship” across items periodically to remind respondents that the items are asking about their internship experience. 88 items were retained after this step.

Step 3: Pre-testing Questions: Ensuring the Questions and Answers Are Meaningful

As the MIES tool was developed and validated in nine countries including countries where English is not the primary language used, the tool was translated into three additional languages (Mandarin Chinese, Vietnamese, and French). The translation processes followed WHO’s guideline for translation which includes forward- and back-translation (7), and were led by collaborators from China, Vietnam and Burundi, respectively. We compared the back-translated version with the original scale items and discussed with the collaborators to ensure that the items were translated correctly.

We then conducted pre-testing to ensure that the items are meaningful to the target population and that the MIES survey could be successfully administered. Pre-testing was conducted in-country collaborators’ proposed mode (online or paper) and in English or translated language and alongside cognitive interviews. Cognitive interviews most specifically focus on face validity and the wording of items, by asking the respondents to verbalise the mental process entailed in providing such answers, to ensure that respondents understand questions as we intended and that respondents can answer in a manner that reflects their experience (8,9). This is especially important for the non-English versions as there could be discrepancies during the translation process.

A total of 19 medical interns were included in cognitive interviews, and the interviews were conducted using a mix of “think aloud” (tell me what you are thinking as you answer this question) and “probing” (what this term X means to you and why you chose that answer). Items were further revised and rephrased at this phase.

Step 4: Survey Administration and Sample Size: Gathering Enough Data from the Right People

The final sample used for analysis was collected from eight study countries including Kenya, Uganda, Burundi, Nigeria, Sierra Leone, Fiji, Vietnam and China as well as an open survey.

The study population eligible for the MIES survey is the current cohort of medical interns or junior medical officers who finished internships in 2018 or after. A variety of rules have been suggested for determining the sample size for the questionnaire survey and scale development: (1) Nunnally suggested for scale development the rule of thumb is 10 participants per item (4,10); (2) Guadagnoli suggested that the actual number is

dependent on the number of items per scale construct and component saturation (the magnitude of component loading), and if there are 10 or more items representing each construct, a sample size of 150 observations should be sufficient to obtain an accurate solution (11). Considering we have 88 items and 3 domains/constructs, we sought to survey at least 150 participants per study country and a total of 1,000 participants due to sample size consideration.

Participants were identified using a mix of snowballing and purposive sampling approaches: for example in China, we advertised the survey through social media only, and in Uganda and Burundi, we visited hospitals where medical interns and junior medical doctors are based and invited them to participate. A filtering question was also included in the survey to ensure participants' eligibility. The survey was self-administered by participants either online (through REDCap, Microsoft Forms or Wenjuanxing [a tool commonly used in China]) or using paper-based questionnaires. Response was considered incomplete and dropped if more than 10% of the scale items were missing – and in our case 77 individuals were dropped.

As of Jan 2023, a total of 1646 complete responses were collected, out of which 113 samples were collected from non-study countries through the open survey, notably South Africa and Tanzania. Only Kenya, Uganda, Vietnam and China had over 150 complete responses (Table 2).

Table 2. MIES final survey sample

Country	Complete response	Percentage as of total sample	Missing 10% of items (dropped sample)
**Uganda**	487	29.59	10
**Kenya**	358	21.75	2
**Vietnam**	177	10.75	2
**China**	160	9.72	7
**Burundi**	120	7.29	46
**Sierra Leone**	98	5.95	3
**Nigeria**	90	5.47	5
**Fiji**	42	2.55	2
**South Africa**	39	2.37	N/A
**Tanzania**	25	1.52	N/A
**Ghana**	17	1.03	N/A
**Philippines**	9	0.55	N/A
**Bangladesh**	6	0.36	N/A
**Namibia**	4	0.24	N/A
**Belize**	3	0.18	N/A
**Malawi**	3	0.18	N/A
**Benin**	2	0.12	N/A
**Ethiopia**	2	0.12	N/A

**Gambia, The**	1	0.06	N/A
**India**	1	0.06	N/A
**Indonesia**	1	0.06	N/A
**Mozambique**	1	0.06	N/A
**Total**	**1,646**		

Step 5: Item Reduction: Ensuring Your Scale Is Parsimonious

We first further explored the level of missingness for each item included in the scale. we calculated how many respondents selected “don’t know/prefer not to say” among all responses for each item. According to the supplementary appendix 5, no item had an over 10% missing rate for the overall sample though for some items there were slightly higher missing rates in countries with small sample sizes. For the rest of the items, we have replaced the missing values with the median of each item. There are different ways to handle missing data in scale development including mean preplacement and multiple imputation (4). We used median instead of mean because most of the items are skewed and mean replacement is valid only if the data is normally distributed; and given the small nature of missing data in our case (all but one items have less than 3% missing rate) we did not use more advanced imputation approaches (12,13).

The objective for item reduction analysis is to ensure that only functional and internally consistent items are included, and we used inter-item and item-total correlations as a technique under the classical test theory (CTT, see Boateng et al. (4)) to select our items. Inter-item correlations measure the extent to which the score of one item is related to scores on all other items, suggesting the extent to which they measure the same construct. Using a cutoff of 0.3 recommended by Boateng et al. (4), six items (Q6, Q17, Q54, Q56, Q67, Q71) were further dropped as they have very low correlations (Table 4).

Step 6: Extraction of Factors: Exploring the Number of Latent Constructs that Fit Your Observed Data

The next step of the scale development process focused on extracting factors and domains of the scale, and we used factor analysis to understand the latent structure of the items. We first calculated the Kaiser-Meyer-Olkin (KMO) measure of sampling adequacy to evaluate the fitness of data for factor analysis, and ran the Bartlett’s test for the correlation matrix. A KMO measure between 0.8-1.0 is considered adequate and for the remaining MIES items the KMO measure was 0.97, and the Bartlett’s test was statistically significant.

The next step is to identify the number of factors or constructs, and there have been a number of different methods to determine the number including scree plots, the variance explained by the factor model and the factor loading pattern (4). We conducted exploratory factor analysis with oblique rotation on the remaining 82 MIES items, six factors had Eigen values exceeding one, explaining 53%, 15%, 10%, 5%, 3% and 3% of the variance respectively (Table 3). An inspection of the Scree plot however, revealed a likely break after the third factor (Figure 1). While there is no absolute correct answer in terms of the number of factors that should be selected, we decided to retain the six-factor solution due to the slightly shorter number of items retained and more intuitive structure.

The three-factor-solution was also explored in the next steps though it did not significantly out-performed the six-factor solution (see Supplementary appendix 4. Three-factor structure for MIES analysis and Supplementary appendix 5. Six-factor structure for MIES analysis).

Table 3. Eigen values for MIES items and retained factors

Factor	Eigen value	Difference	Proportion	Cumulative
Factor1	22.53481	16.01009	0.5319	0.5319
Factor2	6.52472	2.08584	0.1540	0.6859
Factor3	4.43888	2.43607	0.1048	0.7907
Factor4	2.00281	0.54443	0.0473	0.8380
Factor5	1.45838	0.32703	0.0344	0.8724
Factor6	1.13135	0.26705	0.0267	0.8991
Factor7	0.86429	0.02627	0.0204	0.9195
Factor8	0.83802	0.10349	0.0198	0.9393
Factor9	0.73453	0.08261	0.0173	0.9566
Factor10	0.65193	0.09885	0.0154	0.9720

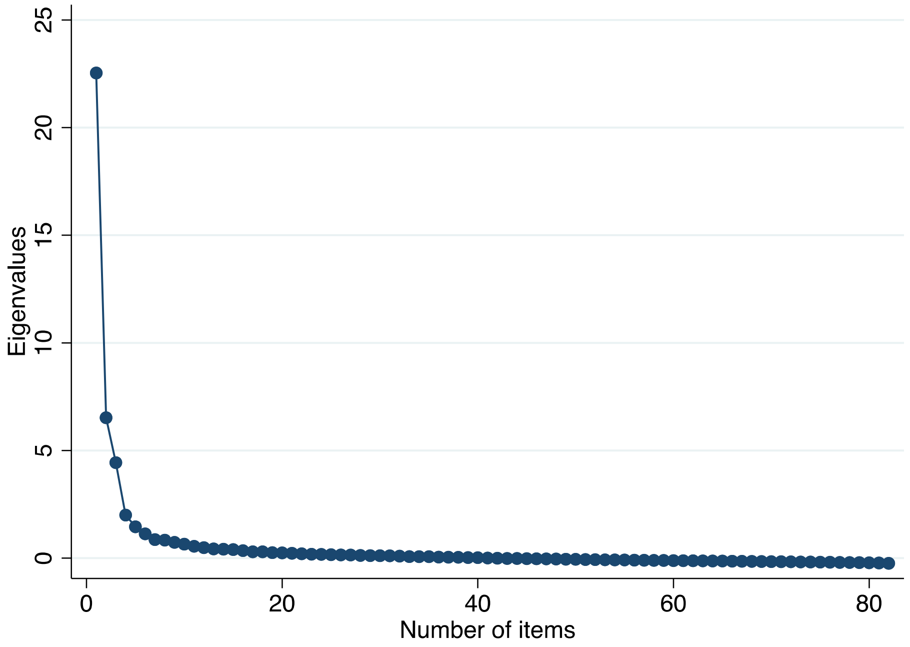

Figure 1. Scree plot for exploratory factor analysis

After restricting the remaining items to a six-factor structure, we further removed items with cross-loading – which we used the 0.4/0.3/0.2 rule, which is items should satisfy (a) load onto their primary factor above 0.40, (b) load onto alternative factors below 0.30, and (c) demonstrate a difference of 0.20 between their primary and alternative factor loadings) (14,15). This removed 32 items across three rounds of testing (Table 4).

In summary, we have identified a six-factor structure for the MIES scale with a total of 50 retained items. After reading the items, we named the six factors (1) clinical learning and supervision; (2) patient safety; (3) stress and burnout; (4) job satisfaction; (5) mental wellbeing; and (6) fairness and discrimination, respectively. Final items and their corresponding factor loading as well as sources are presented in Table 5.

Table 4. Rationale and evidence for dropping MIES items across Step 5 and 6

Step	No.	Item	Rationale
Step 5: Item reduction	Q6	I am able to control irritations in my work and life during my internship.	Low item-rest correlation (0.29)
	Q17	I get satisfaction from being able to help people.	Low item-rest correlation (0.26)
	Q54	I am pre-occupied with administrative work that impeded my ability to learn.	Low item-rest correlation (0.12)
	Q56	There is an informative and comprehensive internship guideline, log book or clinical diary.	Low item-rest correlation (0.27)
	Q67	I am bleeped or called concerning the patients inappropriately during my internship.	Low item-rest correlation (0.30)
	Q71	There is a no-blame culture in my internship hospital.	Low item-rest correlation (0.27)
Step 6: Extraction of factors (Round 1)	Q52	My internship training makes me feel ready to be an independent medical practitioner.	Cross-loading (0.58 on factor 1, 0.32 on factor 3)
	Q62	I have suitable access to careers advice services or advisors during my internship.	Cross-loading (0.38 on factor 1, 0.33 on factor 2)
	Q61	I have good collaboration with other medical practitioners, interns and clinical staff.	Cross-loading (0.37 on factor 1, 0.23 on factor 3)
	Q57	I have the appropriate level of responsibility as a medical intern.	Cross-loading (0.34 on factor 1, 0.20 on factor 2)
	Q55	I had an informative internship induction programme.	Cross-loading (0.33 on factor 1, 0.23 on factor 2)
	Q60	I have the opportunity to provide continuity of care for patients during my internship.	Cross-loading (0.31 on factor 1, 0.29 on factor 2)
	Q65	My work hours are appropriate during my internship.	Cross-loading (0.56 on factor 2, 0.34 on factor 4)
	Q66	My workload is reasonable during my internship.	Cross-loading (0.53 on factor 2, 0.39 on factor 4)
	Q80	I feel physically safe within my internship hospital.	Cross-loading (0.38 on factor 2, 0.18 on factor 5)
	Q40	Time for my own reading, training and education is allocated by the hospital during my internship.	Cross-loading (0.34 on factor 2, 0.22 on factor 1 and 4)
	Q3	I feel confident about my ability to handle my personal problems during my internship.	Cross-loading (0.36 on factor 3, 0.23 on factor 4)
	Q34	I have been affected by the hardship and stress experienced by those patients I help.	Cross-loading (0.61 on factor 4, 0.47 on factor 6)
	Q35	I have felt on edge about various things because of my work helping patients.	Cross-loading (0.59 on factor 4, 0.45 on factor 6)
	Q27	I am not as productive at work because I am losing sleep over work-related traumatic experiences.	Cross-loading (0.45 on factor 4, 0.33 on factor 6)
	Q4	I have felt that things have not gone my way in my life.	Cross-loading (0.45 on factor 4, 0.25 on factor 3)
	Q79	I feel emotionally vulnerable within my internship hospital environment.	Cross-loading (0.41 on factor 5, 0.32 on factor 4)

	Q36	I avoid certain activities or situations because they remind me of frightening experiences of the patients I help.	Cross-loading (0.59 on factor 6, 0.41 on factor 4)
	Q16	I have the thoughts that I would be better off dead or of hurting myself in some way.	Cross-loading (0.37 on factor 6, 0.26 on factor 4)
Step 6: Extraction of factors (Round 2)	Q58	I feel part of a team working here.	Cross-loading (0.43 on factor 1, 0.45 on factor 6)
	Q88	The internship hospital has adequate supply of diagnostics, equipment and medication for my study and work need.	Cross-loading (0.48 on factor 2, 0.48 on factor 6)
	Q75	It is difficult to discuss medical errors in my internship hospital.	Cross-loading (0.37 on factor 2, 0.30 on factor 5)
	Q37	As a result of my helping, I have intrusive, frightening thoughts.	Cross-loading (0.37 on factor 3, 0.22 on factor 6)
	Q18	I feel invigorated and energised after work during my internship.	Cross-loading (0.36 on factor 4, 0.25 on factor 3)
	Q7	I feel that I am on top of things at work.	Cross-loading (0.34 on factor 4, 0.20 on factor 3)
	Q86	There are adequate catering services provided by the internship hospital when I am on call.	Cross-loading (0.55 on factor 6, 0.34 on factor 2)
	Q87	The internship hospital has good internet connection for my study and work need.	Cross-loading (0.48 on factor 6, 0.33 on factor 2)
	Q85	The internship hospital has good quality accommodation for me when on call.	Cross-loading (0.46 on factor 6, 0.29 on factor 2)
	Q63	There are good counselling opportunities for medical interns who fail to complete their training satisfactorily.	Cross-loading (0.32 on factor 6, 0.24 on factor 1)
	Q64	I have a contract of employment or other document that provides information about hours of work during my internship.	Cross-loading (0.315 on factor 6, 0.26 on factor 2)
Step 6: Extraction of factors (Round 3)	Q9	I feel that difficulties at work are piling up so high that I could not overcome them.	Cross-loading (0.39 on factor 3, 0.28 on factor 5)
	Q19	I like my work as a medical intern.	Cross-loading (0.6 on factor 4, 0.32 on factor 3)
	Q13	I feel tired or having little energy during my internship.	Cross-loading (0.44 on factor 5, 0.39 on factor 3)

Table 5. Final items included for the MIES scale

Factor	Question	Item loading	Adapted or new
Factor 1 – Clinical learning and supervision (n=14)<br><br>(Cronbach's alpha: 0.93)	My clinical supervisors are enthusiastic about teaching and supervision.	0.93	PHEEM
	The clinical supervisors provide me with regular feedback.	0.90	
	My clinical supervisors are accessible for teaching and supervision.	0.89	
	My clinical supervisors have good mentoring skills.	0.88	
	The clinical supervisors provide me with feedback on my strengths and weaknesses to ensure my professional development.	0.75	
	My clinical supervisors have good communication skills.	0.74	
	I have enough clinical learning opportunities for my needs during the internship period.	0.68	
	I have good clinical supervision at all times during my internship.	0.67	
	My clinical supervisors encourage me to be an independent learner.	0.63	
	My clinical supervisors have set clear expectations	0.61	
	I am able to participate actively in educational sessions (e.g. continuing medical educations) during my internship.	0.59	
	I have opportunities to acquire the appropriate practical procedures for clinical practice during my internship.	0.58	
	I have access to educational sessions and programmes that are relevant to my needs during my internship	0.58	
	My clinical supervisors promote an atmosphere of mutual respect.	0.55	
Factor 2 – Patient safety (n=10)<br><br>(Cronbach's alpha: 0.90)	There are clear and updated patient safety protocols in the internship hospital.	0.81	New
	Medical errors are handled appropriately in my internship hospital.	0.73	SAQ
	I know the proper channels to direct questions regarding patient safety.	0.72	
	I am encouraged by my colleagues to report any patient safety concerns I may have.	0.70	
	The culture in my internship hospital makes it easy to learn from the errors of others.	0.69	
	I would feel safe being treated as a patient in my internship hospital.	0.68	
	I know the proper channels to direct questions regarding my own safety.	0.65	New
	There are adequate infection prevention and control measures.	0.63	New
	I can report any concern and receive responsive feedback in my internship hospital.	0.63	New
	There are clear clinical protocols and guidelines across all departments in the internship hospital.	0.46	PHEEM
Factor 3 – Stress and burnout (n=10)<br><br>(Cronbach's alpha: 0.90)	I feel overwhelmed because my case workload seems endless during the internship.	0.97	ProQOL
	I feel worn out because of my work as a medical intern.	0.91	
	I feel trapped by my job as a medical intern.	0.71	
	I feel bogged down and held back by the internship hospital.	0.68	
	I am preoccupied by concerns about multiple patients during my internship.	0.67	
	I find it difficult to separate my personal life from my life as a medical intern.	0.63	
	I feel that I am unable to balance my work and personal life during my internship.	0.63	PSS
	I feel nervous and/or stressed because of my internship work.	0.63	
	I find that I could not cope with all the work that I had to do during my internship.	0.45	

	I am angered because of things that were outside of my control.	0.40	
Factor 4 – Job satisfaction (n=7)<br><br>(Cronbach's alpha: 0.88)	I am proud of what I can do to help as a medical intern.	0.93	ProQOL
	I believe I can make a difference through my work.	0.88	
	I believe that I am a success as a medical intern.	0.81	
	I am happy that I chose to do this work.	0.79	
	My internship work makes me feel satisfied.	0.73	
	My ability to keep up with clinical techniques and protocols makes me feel pleased.	0.64	
	My internship experience met my expectation.	0.48	New
Factor 5 – Mental wellbeing (n=5)<br><br>(Cronbach's alpha: 0.84)	I have eating problems, either have poor appetite, or have been overeating.	0.82	PHQ-9
	I have sleeping problems, either have trouble falling or staying asleep, or sleeping too much.	0.76	
	I have trouble concentrating on things either work-related, or outside of my work.	0.66	
	I have little interest or pleasure in doing things that I used to enjoy.	0.63	
	I feel down, depressed, or hopeless because of my internship work.	0.59	
Factor 6 – Fairness and discrimination (n=4)<br><br>(Cronbach's alpha: 0.74)	There is gender discrimination in my internship hospital.	0.73	PHEEM
	There are other forms of discrimination (e.g. ethnicity, religion, tribe, disability) in my internship hospital.	0.73	New
	I get bullied or victimised within my internship hospital.	0.47	New
	I have to perform inappropriate tasks during my internship.	0.44	PHEEM

Step 7: Tests of Dimensionality: Testing if Latent Constructs Are as Hypothesised

We then moved on to examining dimensionality, i.e. to see whether the six-factor-structure is the same across two independent samples, within the same sample at different points or across groups. Due to our sample size we are unable to test it in a different sample, thus we primarily focused on measurement invariance, which is whether the psychometric properties are generalizable across different sub-groups – in our case across different countries.

The first step before testing measurement invariance is running the confirmatory factor analysis on the overall sample again and in each individual country. Table 6 shows the result of the confirmatory factor analysis. Comparative fit index (CFI) measures "the relative improvement in the fit of a researcher's model over that of a baseline model" and ideally should be over 0.95 or 0.90 in a relaxed fit; Root mean square error of approximation (RMSEA) measures "the difference between the examined model and a hypothetical model where every component in the model is related to every other component" and should be below 0.06 or 0.08 in a relaxed fit; Standard root mean square residual (SRMR) is a measure of "the mean absolute correlation residual, the overall difference between the observed and predicted correlations" and also should be below 0.08 or 0.10 in a relaxed fit (4). As shown in Table 6, the overall sample CFI was less than satisfactory while RMSEA and SRMR were satisfactory. The results were similar for the four countries with over 150 respondents' sample sizes.

We further tested the measurement equivalence using multigroup confirmatory factor analysis (Table 7). There are three types of measurement invariance, i.e. configural invariance (same number of factors and pattern of loading), metric invariance (factor loading across groups), scalar invariance (same item intercepts). Due to the less than ideal CFI in the overall sample as examined in the previous step, our model fitted moderately for configural invariance (CFI = 0.86). This suggests some difference in terms of factor loading across different countries. This could be due to various reasons including adequate but still small sample size, cross-loading of items, or others. Nonetheless, this suggests that comparing the results such as aggregate scores across different countries requires extra caution as the scales might perform slightly differently in each country.

Table 6. MIES confirmatory factor analysis result and reliability test

**Country**	**RMSEA (<=0.06 or 0.08)**	**CFI (>=0.95 or 0.90)**	**SRMR (<=0.08 or 0.10)**	**Cronbach's alpha (>0.80)**	**Cronbach's alpha for factor 1**	**Cronbach's alpha for factor 2**	**Cronbach's alpha for factor 3**	**Cronbach's alpha for factor 4**	**Cronbach's alpha for factor 5**	**Cronbach's alpha for factor 6**
Overall	0.049	0.893	0.054	0.95	0.93	0.90	0.90	0.88	0.84	0.74
Multigroup	0.067	0.809	0.086	0.96	0.93	0.91	0.91	0.90	0.85	0.77
Kenya	0.057	0.886	0.063	0.97	0.94	0.93	0.90	0.91	0.88	0.78
Uganda	0.053	0.864	0.062	0.94	0.91	0.89	0.90	0.86	0.82	0.71
Vietnam	0.074	0.801	0.075	0.95	0.95	0.84	0.86	0.91	0.82	0.86
China	0.069	0.831	0.072	0.95	0.95	0.88	0.89	0.91	0.84	0.80

Table 7. MIES multigroup measurement invariance analysis

	χ^2	df	CFI	RMSEA	Comparison	Δ CFI	ΔRMSEA
Unconstrained (configural invariance)	531.733	150	0.856	0.060			
Measurement weights (metric invariance)	356.845	132	0.849	0.061	2 vs 1	0.007	0.001
Structural covariance (scalar invariance)	732.637	131	0.794	0.070	3 vs 2	0.055	0.009

Step 8: Tests of Reliability: Establishing if Responses Are Consistent When Repeated

We calculated Cronbach's alpha to assess the internal consistency of the scale items, which is one commonly used reliability criteria. As shown in Table 6, Cronbach's alpha for the overall final scale items was 0.95 and ranged from 0.74 to 0.93 for the six identified factors. The scale- and factor-specific alpha results were similar for most countries. Due to data limitations, we were unable to examine the test-retest reliability.

Step 9: Tests of Validity: Ensuring You Measure the Latent Dimension You Intended

We examined the validity of MIES in line with Cook's recommendation, which defined validity as "the degree to which a score can be interpreted as representing the intended underlying construct" that incorporated five different aspects, i.e. content validity, response process, internal structure, relations to other variables and consequences (16).

Content validity of MIES was ensured as the item development process included a scoping review to identify relevant tools, as well as content validity discussions with both the target population and an expert panel. Response process refers to how well the respondents' response aligns with the intended construct, and in my case we used cognitive interviews as part of pilot testing to ensure that respondents understand the items as we designed. Evidence on the internal structure of the scale derived from internal consistency and factor structure analysis; We do not yet have evidence on relations to other variables and consequences for the MIES scale, though the tool could be used in the future to identify hospitals that trained interns with poorer internship experience and further improve internship training environment.

Supplementary appendix 2. Changes to MIES items from original tools to post-content validity discussion

Left hand side items in black are original tool items, and items on the right hand in red are items changed after discussions with target population.

The questions in this section will ask you about your feelings and thoughts during your internship year. **Please reflect on your whole internship experience in all department rotations rather than your current department rotation**. Please circle how often you felt or thought a certain way.

5 = Very Often	4 = Often	3 = Sometimes	2 = Rarely	1 = Never

Domain A. Stress

#	During your internship year, how often have you been bothered by any of the following feelings or thoughts?	During your internship year, how often have you been bothered by any of the following feelings or thoughts?
A1	I am upset because of something that happened unexpectedly.	I am upset because of unexpected work-related incidents.
A2	I feel that I am unable to control the important things in my life.	I feel that I am unable to balance my work and personal life.
A3	I feel nervous and “stressed”.	I feel nervous and/or “stressed” because of my work.
A4	I feel confident about my ability to handle my personal problems.	I feel confident about my ability to handle my personal problems. (No change)
A5	I feel that things were not going my way.	I feel that things were not going my way. (no change)
A6	I find that I could not cope with all the things that I had to do.	I find that I could not cope with all the work that I had to do.
A7	I am able to control irritations in my life.	I am able to control irritations in my work and life.
A8	I feel that I am on top of things.	I feel that I am on top of things. (no change)
A9	I am angered because of things that were outside of my control.	I am angered because of things that were outside of my control. (No change)
A10	I feel that difficulties are piling up so high that I could not overcome them.	I feel that difficulties at work are piling up so high that I could not overcome them.

Domain B. Depression

#	**During your internship year, how often do you experience these feelings or thoughts?**	**During your internship year, how often do you experience these feelings or thoughts?**
B1	I have little interest or pleasure in doing things.	I have little interest or pleasure in doing things that I used to enjoy.
B2	I feel down, depressed, or hopeless.	I feel down, depressed, or hopeless because of my work.
B3	I have trouble falling or staying asleep, or sleeping too much.	I have trouble falling or staying asleep, or sleeping too much. (No change)
B4	I feel tired or having little energy.	I feel tired or having little energy. (No change)
B5	I have poor appetite, or overeating.	I have poor appetite, or overeating. (No change)
B6	I feel bad about myself—or that I am a failure or have let myself or my family down.	I feel bad about myself—or that I am a failure or have let myself or my family down. (No change)
B7	I have trouble concentrating on things, such as reading newspaper or watching television.	I have trouble concentrating on things either work-related, or outside of my work.
B8	I move or speak so slowly that other people could have noticed? Or the opposite—being so fidgety or restless that I move around a lot more than usual?	~~I move or speak so slowly that other people could have noticed? Or the opposite—being so fidgety or restless that I move around a lot more than usual?~~
B9	I have the thoughts that I would be better off dead or of hurting myself in some way.	I have the thoughts that I would be better off dead or of hurting myself in some way. (No change)

When you help people, you have direct contact with their lives. As you may have found, your compassion for those you help can affect you in positive and negative ways. Below are some questions about your experiences, both positive and negative, as a helper. **Consider each of the following questions about you and your work situation during your whole internship**. Select the number that honestly reflects how frequently you experienced these things during your internship year.

Domain C. Satisfaction

#	**During your internship year, how often do you experience these things?**	**During your internship year, how often do you experience these things?**
C1	I am happy.	I am happy.
C2	I get satisfaction from being able to help people.	I get satisfaction from being able to help people. (No change)
C3	I feel invigorated after working with those I help.	I feel invigorated after work.
C4	I like my work as a helper.	I like my work as a helper during internship.

C5	I am pleased with how I am able to keep up with helping techniques and protocols.	I am pleased with how I am able to keep up with clinical techniques and protocols.
C6	My work makes me feel satisfied.	My work makes me feel satisfied.
C7	I have happy thoughts and feelings about those I help and how I could help them.	~~I have happy thoughts and feelings about those I help and how I could help them.~~
C8	I believe I can make a difference through my work.	I believe I can make a difference through my work.
C9	I am proud of what I can do to help.	I am proud of what I can do to help.
C10	I have thoughts that I am a "success" as a helper.	I believe that I am a "success" as a medical intern.
C11	I am happy that I chose to do this work.	I am happy that I chose to do this work. (No change)
C12		My internship experience met my expectation
D5		I am the person I always wanted to be.

Domain D. Burnout

#	**During your internship year, how often do you experience these things?**	**During your internship year, how often do you experience these things?**
D1	I feel connected to others.	I feel connected to others.
D2	I am not as productive at work because I am losing sleep over traumatic experiences of a person I help.	I am not as productive at work because I am losing sleep over work-related traumatic experiences.
D3	I feel trapped by my job as a helper.	I feel trapped by my job as a medical intern.
D4	I have beliefs that sustain me.	I have beliefs that sustain me.
~~D5~~	I am the person I always wanted to be.	(Moved to domain C)
D6	I feel worn out because of my work as a helper.	I feel worn out because of my work as a medical intern.
D7	I feel overwhelmed because my case workload seems endless.	I feel overwhelmed because my case workload seems endless. (No change)
D8	I feel "bogged down" by the system.	I feel "bogged down" by the hospital.
D9	I am a very caring person.	~~I am a very caring person.~~

Domain E. Compassion fatigue

#	During your internship year, how often do you experience these things?	During your internship year, how often do you experience these things?
E1	I am preoccupied with more than one person I help.	I am preoccupied with more than one patient I am approaching and helping.
E2	I jump or am startled by unexpected sounds.	~~I jump or am startled by unexpected sounds.~~
E3	I find it difficult to separate my personal life from my life as a helper.	I find it difficult to separate my personal life from my life as a medical intern.
E4	I think that I might have been affected by the traumatic stress of those I help.	I think that I might have been affected by the traumatic stress of those patient I help.
E5	Because of my helping, I have felt "on edge" about various things.	Because of my helping at work, I have felt "on edge" about various things.
E6	I feel depressed because of the traumatic experiences of the people I help.	I feel depressed because of the traumatic experiences of the patient I help.
E7	I feel as though I am experiencing the trauma of someone I have helped.	I feel as though I am experiencing the trauma of the patient I have helped.
E8	I avoid certain activities or situations because they remind me of frightening experiences of the people I help.	I avoid certain activities or situations because they remind me of frightening experiences of the patient I help.
E9	As a result of my helping, I have intrusive, frightening thoughts.	As a result of my helping, I have intrusive, frightening thoughts. (No change)
E10	I can't recall important parts of my work with trauma victims.	I can't recall important parts of my work with trauma victims. (No change(ß

5 = Strongly agree	4 = Agree	3 = Neutral	2 = Disagree	1 = Strongly disagree

This section as your perception of the internship training centre you are currently affiliated with or previously affiliated with. Please circle to what extent do you agree with these statements.

Domain F. Teaching and supervision

#	To what extent do you agree with the following statements?	To what extent do you agree with the following statements?
F1	My clinical teachers set clear expectations	My clinical teachers have set clear expectations
F2	I have good clinical supervision at all times	I have good clinical supervision at all times (No change)
F3	I have protected educational time in this post	I have allocated time for training and education during internship.

F4	My clinical teachers have good communication skills	My clinical teachers have good communication skills (No change)
F5	I am able to participate actively in educational events	I am able to participate actively in educational sessions
F6	My clinical teachers are enthusiastic	My clinical teachers are enthusiastic about teaching and supervision
~~F7~~	There is access to an educational programme relevant to my needs	There is access to an educational programme relevant to my needs (no change)
F8	I get regular feedback from seniors	The clinical teachers provide me with regular feedback
F9	My clinical teachers are well organised	My clinical teachers are well organised (No change)
F10	I have enough clinical learning opportunities for my needs	I have enough clinical learning opportunities for my needs (No change)
F11	My clinical teachers have good teaching skills	My clinical teachers have good teaching skills (No change)
F12	My clinical teachers are accessible	My clinical teachers are accessible (No change)
F13	Senior staff utilize learning opportunities effectively	My clinical teachers and other staff utilize different learning opportunities effectively
F14	My clinical teachers encourage me to be an independent learner	My clinical teachers encourage me to be an independent learner (No change)
F15	The clinical teachers provide me with good feedback on my strengths and weaknesses	The clinical teachers provide me with feedback on my strengths and weaknesses
F16	My clinical teachers have good mentoring skills	My clinical teachers have good mentoring skills (No change)
F17	I have opportunities to acquire the appropriate practical procedures for my grade	I have opportunities to acquire the appropriate practical procedures for my clinical practice
F18	The training in this post makes me feel ready to be a medical officer	My internship training makes me feel ready to be an independent medical practitioner
F19	My clinical teachers promote an atmosphere of mutual respect	My clinical teachers promote an atmosphere of mutual respect (No change)
F20		I am pre-occupied with administrative work that impeded my ability to learn

Domain G. Organizational support

#	**To what extent do you agree with the following statements?**	**To what extent do you agree with the following statements?**

G1	I had an informative induction programme	I had an informative induction programme (No change)
G2	There is an informative Internship Guideline and Log book	There is an informative and comprehensive internship guideline, log book or clinical diary
G3	I have the appropriate level of responsibility in this post	I have the appropriate level of responsibility as a medical intern
G4	I feel part of a team working here	I feel part of a team working here (No change)
G5	There are clear clinical protocols in this post	There are clear clinical protocols
G6	I have the opportunity to provide continuity of care	I have the opportunity to provide continuity of care (No change)
G7	I have good collaboration with other doctors in my grade	I have good collaboration with other medical practitioners, interns and clinical staff
G8	I have suitable access to careers advice	I have suitable access to careers advice services and/or advisors
G9	There are good counselling opportunities for junior doctors who fail to complete their training satisfactorily	There are good counselling opportunities for medical interns who fail to complete their training satisfactorily

Domain H. Work condition and safety climate

#	To what extent do you agree with the following statements?	To what extent do you agree with the following statements?
H1	I have a contract of employment that provides information about hours of work	I have a contract of employment or other document that provides information about hours of work
H2	My hours conform to the General Wages Order	My work hours is appropriate
H3	My workload in this job is fine	My workload in this job is fine (No change)
H4	I am bleeped inappropriately	I am bleeped or called concerning the patients inappropriately
H5	I have to perform inappropriate tasks	I have to perform inappropriate tasks
H6	There is sex discrimination in this post	There is gender discrimination
H7	There are other forms of discrimination in this post (other than sex discrimination)	There are other forms of discrimination (e.g. ethnicity, religion, tribe, disability)
H9	There is a no-blame culture in this post	There is a no-blame culture
H10	I get a lot of enjoyment out of my present job	~~I get a lot of enjoyment out of my present job~~
J1	I would feel safe being treated here as a patient	I would feel safe being treated here as a patient (No change)

J2	Medical errors are handled appropriately in my clinical area	Medical errors are handled appropriately here (No change)
J3	I know the proper channels to direct questions regarding patient safety in my clinical area	I know the proper channels to direct questions regarding patient safety (No change)
J4	I receive appropriate feedback about my performance	~~I receive appropriate feedback about my performance~~
J5	In my clinical area, it is difficult to discuss errors	It is difficult to discuss errors (No change)
J6	I am encouraged by my colleagues to report any patient safety concerns I may have	I am encouraged by my colleagues to report any patient safety concerns I may have (No change)
J7	The culture in my clinical area makes it easy to learn from the errors of others	The culture in my clinical area makes it easy to learn from the errors of others (No change)
J8		I know the proper channels to direct questions regarding my own safety
J9		I feel emotionally vulnerable within the hospital environment
H8	I feel physically safe within the hospital environment	I feel physically safe within the hospital environment (No change)
J10		I get bullied or victimised within the hospital environment
J11		There are clear and updated patient safety protocols (No change)
J12		There is adequate infection prevention and control measures
J13		I can report any concern and receive responsive feedback.

Domain I. Physical and financial resource

#	To what extent do you agree with the following statements?	To what extent do you agree with the following statements?
I1	This hospital has good quality accommodation for me, especially when on call	The internship hospital has good quality accommodation for me when on call (No change)
I2	There are adequate catering facilities when I am on call	There are adequate catering services provided by the hospital when I am on call
I3	This hospital has good internet connection for my study and work need	This hospital has good internet connection for my study and work need (No change)
I4	This hospital has adequate amenities and equipment for my study and work need	This hospital has adequate supply of diagnostics, equipment and medication for my study and work need

I5	This hospital has adequate supply of diagnostics and medication for my study and work need	~~This hospital has adequate supply of medication for my study and work need~~
I6	I receive acceptable pay and remuneration for my study and work need	I receive acceptable pay and remuneration for my study and work need (No change)

Supplementary appendix 3. Expert panel members involved in scale evaluation

Name	Affiliation	Expertise
Kun Tang	Tsinghua University, China	Internship
Nathanael Sirili	Muhimbili University of Health and Allied Sciences, Tanzania	Internship
Raymond Tweheyo	Department of Health Policy Planning and Management, School of Public Health, Makerere University, Uganda	Scale development
Mike English	University of Oxford, UK	Internship & scale development
Xiaoming Xu	University Medical Center Groningen, Netherlands	Scale development
Okello Tom Richard	Lira university, Uganda	Internship
Phùng Khánh Lâm	University of Medicine and Pharmacy at Ho Chi Minh City, Vietnam	Scale development
Thai Thanh Truc	Department of Medical Statistics and Informatics, University of Medicine and Pharmacy at Ho Chi Minh City, Vietnam	Internship & scale development
Richard Summers	Swansea University, UK	Scale development
Shobhana Nagraj	University of Oxford, UK	Internship & scale development
Sulaiman Jalloh	Ola During Children's Hospital , Sierra Leone	Internship
Nadine Misago	Health Healing Network Burundi, Burundi	Internship & scale development
Desire Habonimana	University of Burundi, Burundi	Internship & scale development
Daniel Mbhthia	KEMRI-Wellcome Trust, Kenya	Internship

Supplementary appendix 4. English, French, Mandarin and Vietnamese version of the 88-version MIES scale

The questions in this section will ask you about your feelings and thoughts during your internship year(s). It also ask about your experiences, both positive and negative, as a helper: When you help people, you have direct contact with their lives. As you may have found, your compassion for those you help can affect you in positive and negative ways.
Please reflect on your whole internship experience in all department rotations rather than your current department rotation.
Please circle how often you felt or thought a certain way.

	**During your internship year, how often do you experience these feelings or thoughts?**	**5 = Very Often**	**4 = Often**	**3 = Someti mes**	**2 = Rarely**	**1 = Never**	**Don't know/ Prefer not to say**
Q1	I feel that I am unable to balance my work and personal life during my internship.	5	4	3	2	1	X
Q2	I feel nervous and/or stressed because of my internship work.	5	4	3	2	1	X
Q3	I feel confident about my ability to handle my personal problems during my internship.	5	4	3	2	1	X
Q4	I have felt that things have not gone my way in my life.	5	4	3	2	1	X
Q5	I find that I could not cope with all the work that I had to do during my internship.	5	4	3	2	1	X
Q6	I am able to control irritations in my work and life during my internship.	5	4	3	2	1	X
Q7	I feel that I am on top of things at work.	5	4	3	2	1	X
Q8	I am angered because of things that were outside of my control.	5	4	3	2	1	X
Q9	I feel that difficulties at work are piling up so high that I could not overcome them.	5	4	3	2	1	X
Q10	I have little interest or pleasure in doing things that I used to enjoy.	5	4	3	2	1	X
Q11	I feel down, depressed, or hopeless because of my internship work.	5	4	3	2	1	X
Q12	I have sleeping problems, either have trouble falling or staying asleep, or sleeping too much.	5	4	3	2	1	X
Q13	I feel tired or having little energy during my internship.	5	4	3	2	1	X

Q14	I have eating problems, either have poor appetite, or have been overeating.	5	4	3	2	1	X
	**During your internship year, how often do you experience these feelings or thoughts?**	**5 = Very Often**	**4 = Often**	**3 = Sometimes**	**2 = Rarely**	**1 = Never**	**Don't know/ Prefer not to say**
Q15	I have trouble concentrating on things either work-related, or outside of my work.	5	4	3	2	1	X
Q16	I have the thoughts that I would be better off dead or of hurting myself in some way.	5	4	3	2	1	X
Q17	I get satisfaction from being able to help people.	5	4	3	2	1	X
Q18	I feel invigorated and energized after work during my internship.	5	4	3	2	1	X
Q19	I like my work as a medical intern.	5	4	3	2	1	X
Q20	My ability to keep up with clinical techniques and protocols makes me feel pleased.	5	4	3	2	1	X
Q21	My internship work makes me feel satisfied.	5	4	3	2	1	X
Q22	I believe I can make a difference through my work.	5	4	3	2	1	X
Q23	I am proud of what I can do to help as a medical intern.	5	4	3	2	1	X
Q24	I believe that I am a success as a medical intern.	5	4	3	2	1	X
Q25	I am happy that I chose to do this work.	5	4	3	2	1	X
Q26	My internship experience met my expectation.	5	4	3	2	1	X
Q27	I am not as productive at work because I am losing sleep over work-related traumatic experiences.	5	4	3	2	1	X
Q28	I feel trapped by my job as a medical intern.	5	4	3	2	1	X
Q29	I feel worn out because of my work as a medical intern.	5	4	3	2	1	X
Q30	I feel overwhelmed because my case workload seems endless during the internship.	5	4	3	2	1	X
Q31	I feel bogged down and held back by the internship hospital.	5	4	3	2	1	X
Q32	I am preoccupied by concerns about multiple patients during my internship.	5	4	3	2	1	X

Q33	I find it difficult to separate my personal life from my life as a medical intern.	5	4	3	2	1	X
	**During your internship year, how often do you experience these feelings or thoughts?**	**5 = Very Often**	**4 = Often**	**3 = Sometimes**	**2 = Rarely**	**1 = Never**	**Don't know/ Prefer not to say**
Q34	I have been affected by the hardship and stress experienced by those patients I help.	5	4	3	2	1	X
Q35	I have felt on edge about various things because of my work helping patients.	5	4	3	2	1	X
Q36	I avoid certain activities or situations because they remind me of frightening experiences of the patients I help.	5	4	3	2	1	X
Q37	As a result of my helping, I have intrusive, frightening thoughts.	5	4	3	2	1	X

This section asks your perception of the internship training centre(s) you are currently affiliated with or previously affiliated with. It also asks your perception of the clinical supervisors, i.e. those who provided teaching and supervision for you during the internship period.
Please circle to what extent do you agree with these statements.

	**To what extent do you agree with the following statements?**	**5 = Strongly agree**	**4 = Agree**	**3 = Neutral**	**2 = Disagree**	**1 = Strongly disagree**	**Don't know/ Prefer not to say**
Q38	My clinical supervisors have set clear expectations	5	4	3	2	1	X
Q39	I have good clinical supervision at all times during my internship.	5	4	3	2	1	X
Q40	Time for my own reading, training and education is allocated by the hospital during my internship.	5	4	3	2	1	X
Q41	My clinical supervisors have good communication skills.	5	4	3	2	1	X
Q42	I am able to participate actively in educational sessions (e.g. continueing medical educations) during my internship.	5	4	3	2	1	X
Q43	My clinical supervisors are enthusiastic about teaching and supervision.	5	4	3	2	1	X

Q44	I have access to educational sessions and programmes that are relevant to my needs during my internship	5	4	3	2	1	X
	**To what extent do you agree with the following statements?**	**5 = Strongly agree**	**4 = Agree**	**3 = Neutral**	**2 = Disagree**	**1 = Strongly disagree**	**Don't know/ Prefer not to say**
Q45	The clinical supervisors provide me with regular feedback.	5	4	3	2	1	X
Q46	I have enough clinical learning opportunities for my needs during the internship period.	5	4	3	2	1	X
Q47	My clinical supervisors are accessible for teaching and supervision.	5	4	3	2	1	X
Q48	My clinical supervisors encourage me to be an independent learner.	5	4	3	2	1	X
Q49	The clinical supervisors provide me with feedback on my strengths and weaknesses to ensure my professional development.	5	4	3	2	1	X
Q50	My clinical supervisors have good mentoring skills.	5	4	3	2	1	X
Q51	I have opportunities to acquire the appropriate practical procedures for clinical practice during my internship.	5	4	3	2	1	X
Q52	My internship training makes me feel ready to be an independent medical practitioner.	5	4	3	2	1	X
Q53	My clinical supervisors promote an atmosphere of mutual respect.	5	4	3	2	1	X
Q54	I am pre-occupied with administrative work that impeded my ability to learn.	5	4	3	2	1	X
Q55	I had an informative internship induction programme.	5	4	3	2	1	X
Q56	There is an informative and comprehensive internship guideline, log book or clinical diary.	5	4	3	2	1	X
Q57	I have the appropriate level of responsibility as a medical intern.	5	4	3	2	1	X
Q58	I feel part of a team working here.	5	4	3	2	1	X

Q59	There are clear clinical protocols and guidelines across all departments in the internship hospital.	5	4	3	2	1	X
Q60	I have the opportunity to provide continuity of care for patients during my internship.	5	4	3	2	1	X
Q61	I have good collaboration with other medical practitioners, interns and clinical staff.	5	4	3	2	1	X
	**To what extent do you agree with the following statements?**	**5 = Strongly agree**	**4 = Agree**	**3 = Neutral**	**2 = Disagree**	**1 = Strongly disagree**	**Don't know/ Prefer not to say**
Q62	I have suitable access to careers advice services or advisors during my internship.	5	4	3	2	1	X
Q63	There are good counselling opportunities for medical interns who fail to complete their training satisfactorily.	5	4	3	2	1	X
Q64	I have a contract of employment or other document that provides information about hours of work during my internship.	5	4	3	2	1	X
Q65	My work hours are appropriate during my internship.	5	4	3	2	1	X
Q66	My workload is reasonable during my internship.	5	4	3	2	1	X
Q67	I am bleeped or called concerning the patients inappropriately during my internship.	5	4	3	2	1	X
Q68	I have to perform inappropriate tasks during my internship.	5	4	3	2	1	X
Q69	There is gender discrimination in my internship hospital.	5	4	3	2	1	X
Q70	There are other forms of discrimination (e.g. ethnicity, religion, tribe, disability) in my internship hospital.	5	4	3	2	1	X
Q71	There is a no-blame culture in my internship hospital.	5	4	3	2	1	X
Q72	I would feel safe being treated as a patient in my internship hospital.	5	4	3	2	1	X
Q73	Medical errors are handled appropriately in my internship hospital.	5	4	3	2	1	X

Q74	I know the proper channels to direct questions regarding patient safety.	5	4	3	2	1	X
Q75	It is difficult to discuss medical errors in my internship hospital.	5	4	3	2	1	X
Q76	I am encouraged by my colleagues to report any patient safety concerns I may have.	5	4	3	2	1	X
Q77	The culture in my internship hospital makes it easy to learn from the errors of others.	5	4	3	2	1	X
	**To what extent do you agree with the following statements?**	**5 = Strongly agree**	**4 = Agree**	**3 = Neutral**	**2 = Disagree**	**1 = Strongly disagree**	**Don't know/ Prefer not to say**
Q78	I know the proper channels to direct questions regarding my own safety.	5	4	3	2	1	X
Q79	I feel emotionally vulnerable within my internship hospital environment	5	4	3	2	1	X
Q80	I feel physically safe within my internship hospital.	5	4	3	2	1	X
Q81	I get bullied or victimized within my internship hospital.	5	4	3	2	1	X
Q82	There are clear and updated patient safety protocols in the internship hospital.	5	4	3	2	1	X
Q83	There are adequate infection prevention and control measures.	5	4	3	2	1	X
Q84	I can report any concern and receive responsive feedback in my internship hospital.	5	4	3	2	1	X
Q85	The internship hospital has good quality accommodation for me when on call.	5	4	3	2	1	X
Q86	There are adequate catering services provided by the internship hospital when I am on call.	5	4	3	2	1	X
Q87	The internship hospital has good internet connection for my study and work need.	5	4	3	2	1	X
Q88	The internship hospital has adequate supply of diagnostics, equipment and medication for my study and work need.	5	4	3	2	1	X

Dans cette section, des questions sont posées à propos de vos sentiments et vos pensées au cours de votre (vos) année(s) de stage d'internat. Des questions à propos de votre expérience positive et négative, en tant qu'assistant sont aussi posées: lorsque vous aidez les gens, vous avez un contact direct avec leur vie. Comme vous l'avez peut-être constaté, votre compassion pour ceux que vous aidez peut vous affecter de manière positive et négative. **Veuillez réfléchir à l'ensemble de votre expérience de stage dans tous les département plutôt qu'à votre département actuel. Veuillez encercler à quelle frequence vous avez ressenti ou pensé d'une certaine manière.**

	**Au cours de votre période de stage, à quelle fréquence ressentez-vous ces sentiments ou pensées ?**	**5 = Très souvent**	**4 = Souvent**	**3 = Parfois**	**2 = Rarement**	**1 = Jamais**	**Ne sait pas/Neutre**
Q1	Je me sens incapable d'équilibrer mon travail et ma vie personnelle pendant mon stage d'internat	5	4	3	2	1	X
Q2	Je me sens nerveux et/ou stressé à cause de mon travail de stage	5	4	3	2	1	X
Q3	J'ai confiance en ma capacité de gérer mes problèmes personnels pendant mon stage	5	4	3	2	1	X
Q4	J'ai senti que les choses n'allaient pas au sens de ma vie	5	4	3	2	1	X
Q5	Je trouve que je ne pouvais pas faire face à tout le travail que j'avais à faire pendant mon stage.	5	4	3	2	1	X
Q6	Je suis capable de contrôler les irritations dans mon travail et ma vie pendant mon stage.	5	4	3	2	1	X
Q7	Je sens que je suis à mesure de mieux faire au travail	5	4	3	2	1	X
Q8	Je suis en colère à cause des choses qui échappaient à mon contrôle.	5	4	3	2	1	X
Q9	Je sens que les difficultés au travail s'accumulent beaucoup que je ne pourrais pas les surmonter.	5	4	3	2	1	X
Q10	J'ai peu d'intérêt ou de plaisir à faire des choses que j'aimais.	5	4	3	2	1	X
Q11	Je me sens épuisé, déprimé ou désespéré à cause de mon travail de stage.	5	4	3	2	1	X
Q12	J'ai des problèmes de sommeil, j'ai du mal à m'endormir ou à rester endormi, ou à trop dormir.	5	4	3	2	1	X
Q13	Je me sens fatigué ou j'ai peu d'énergie pendant mon stage	5	4	3	2	1	X
Q14	J'ai des problèmes d'alimentation, j'ai un manque d'appétit ou j'ai trop mangé.	5	4	3	2	1	X
Q15	J'ai du mal à me concentrer sur des choses liées au travail ou en dehors de mon travail.	5	4	3	2	1	X

Q16	J'ai l'impression que je ferais mieux de mourir ou de me blesser d'une manière ou d'une autre.	5	4	3	2	1	X
	**Au cours de votre période de stage, à quelle fréquence ressentez-vous ces sentiments ou pensées ?**	**5 = Très souven t**	**4 = Souven t**	**3 = Parfois**	**2 = Rareme nt**	**1 = Jamais**	**Ne sait pas/Ne utre**
Q17	Je suis satisfait de pouvoir aider les gens.	5	4	3	2	1	X
Q18	Je me sens en forme après le travail pendant mon stage	5	4	3	2	1	X
Q19	J'aime mon travail comme médecin stagiaire.	5	4	3	2	1	X
Q20	Ma capacité de suivre les techniques et les protocoles cliniques me rend satisfait.	5	4	3	2	1	X
Q21	Mon travail de stage me satisfait.	5	4	3	2	1	X
Q22	Je crois que je peux faire la différence à travers mon travail.	5	4	3	2	1	X
Q23	Je suis fier de ce que je peux faire pour aider en tant que médecin stagiaire.	5	4	3	2	1	X
Q24	Je crois que j'ai réussi en tant que stagiaire en médecine.	5	4	3	2	1	X
Q25	Je suis content d'avoir choisi de faire ce travail.	5	4	3	2	1	X
Q26	Mon expérience de stage a satisfait à mes attentes	5	4	3	2	1	X
Q27	Je ne suis pas aussi productif au travail parce que je perds le sommeil à cause d'expériences traumatisantes liées au travail.	5	4	3	2	1	X
Q28	Je me sens piéger par mon travail comme médecin stagiaire.	5	4	3	2	1	X
Q29	Je me sens épuisé à cause de mon travail comme médecin stagiaire.	5	4	3	2	1	X
Q30	Je me sens dépassé parce que le cas de ma charge de travail semble interminable pendant le stage.	5	4	3	2	1	X
Q31	Je me sens blocqué et retenu par l'hôpital de mon stage.	5	4	3	2	1	X
Q32	Je suis préoccupé par les inquiétudes concernant plusieurs patients pendant mon stage	5	4	3	2	1	X
Q33	J'ai du mal à séparer ma vie personnelle de ma vie de médecin stagiaire.	5	4	3	2	1	X
Q34	J'ai été affectée par les difficultés et le stress vécus par les patient que j'aide	5	4	3	2	1	X

Q35	Je me suis senti nerveux concernant différentes choses à cause de mon travail d'aider les patients.	5	4	3	2	1	X
Q36	J'évite certaines activités ou situations parce que elles me rappellent des expériences effrayantes des patients que j'aide	5	4	3	2	1	X
Q37	A cause de mon aide, j'ai des pensées intrusives et effrayantes.	5	4	3	2	1	X

Cette section demande votre perception de l'hôpital d'affiliation actuelle ou ancienne pour votre stage d'internat. Elle demande aussi votre perception des superviseurs cliniques, exemple ceux qui vous enseignent et vous supervisent pendant la période de stages.
Veuillez encercler à quelle point vous êtes d'accord avec ces affirmations

	**A quelle point êtes -vous d'accord avec les affirmations suivantes?**	**5 = Très d'accord**	**4 = En accord**	**3 = Neutre**	**2 = En desaccord**	**1 = Très en desaccord**	**Ne sait pas/Ne dit rien**
Q38	Mes superviseurs cliniques ont défini des attentes claires	5	4	3	2	1	X
Q39	J'ai une bonne supervision Clinique pendant tout le temps de mes stages d'internat.	5	4	3	2	1	X
Q40	Le temps pour ma propre lecture, formation et éducation est alloué par l'hôpital pendant mon stage.	5	4	3	2	1	X
Q41	Mes superviseurs cliniques ont de bonnes compétences en communication	5	4	3	2	1	X
Q42	Je suis capable de participer activement à des sessions éducatives pendant mon stage (par exemple, formation médicale continue)	5	4	3	2	1	X
Q43	Mes superviseurs cliniques sont enthousiastes à propos de l'enseignement et de la supervision	5	4	3	2	1	X
Q44	J'ai access à des session et programmes éducatif relatifs a mes besoins pendant mon stage	5	4	3	2	1	X
Q45	Les superviseurs cliniques me donnent un retour d'information régulier	5	4	3	2	1	X
Q46	J'ai des opportunités suffisantes d'apprentissage clinique pour mes besoins pendant la période de stage	5	4	3	2	1	X

Q47	Mes superviseurs cliniques sont accessibles pour l'enseignement et la supervision.	5	4	3	2	1	X
Q48	Mes superviseurs cliniques m'encouragent d' être un apprenant indépendant	5	4	3	2	1	X
Q49	Les superviseurs cliniques me donnent un retour d'information concernant mes forces et faiblesses pour assurer mon développement professionnel.	5	4	3	2	1	X
Q50	Mes superviseurs cliniques ont de bonnes compétences de mentorat	5	4	3	2	1	X
Q51	J'ai les opportunités d'acquérir les procédures pratiques appropriées pour la pratique clinique pendant mon stage	5	4	3	2	1	X
	**A quelle mesure êtes-vous d'accord avec les affirmations suivantes ?**	**5 = Très d'accord**	**4 = En accord**	**3 = Neutre**	**2 = En desaccord**	**1 = Très en desaccord**	**Ne sait pas/Ne dit rien**
Q52	Ma formation de stage d'internat me fait sentir pret à être un medecin praticien indépendant	5	4	3	2	1	X
Q53	Mes superviseurs cliniques promeuvent un atmosphère de respect mutuel	5	4	3	2	1	X
Q54	Je suis préoccupé par le travail administratif qui entrave ma capacité d'apprendre	5	4	3	2	1	X
Q55	Jai eu un programme informatif d'initiation au stage	5	4	3	2	1	X
Q56	Il existe un guide informatif et complet de stage, un journal de bord ou un journal clinique	5	4	3	2	1	X
Q57	J'ai un niveau approprié de responsibility en tant que medecin stagiaire	5	4	3	2	1	X
Q58	Je me sens etre partie prenante d'une équipe qui travaille ici	5	4	3	2	1	X
Q59	Il existe des protocoles et des directives cliniques clairs dans tous les services de l'hôpital d'internat. J'ai l'opportunité d'assurer la continuité des soins aux patients durant mon internat.	5	4	3	2	1	X
Q60	J'ai l'opportunité d'assurer la continuité des soins pendant mon stage	5	4	3	2	1	X

Q61	J'ai une bonne collaboration avec les autres médecins praticiens internes et le staff clinique	5	4	3	2	1	X
Q62	J'ai un access adapté à des services de conseil en carrière ou à des conseillers pendant mon stage	5	4	3	2	1	X
Q63	Ils existent de bonnes opportunités de conseil pour les internes en médecine qui echouent de terminer leur formation avec succèss	5	4	3	2	1	X
Q64	Jai un contrat de travail ou un autre document qui fournit des informations sur les heures de travail pendant mon stage	5	4	3	2	1	X
Q65	Les heures de travail pendant mon stage sont appropriées	5	4	3	2	1	X
Q66	Ma charge de travail est acceptable et raisonnable pendant mes stages	5	4	3	2	1	X
Q67	Je reçois un bip ou un appel concernant les patients de manière inappropriée pendant mon stage	5	4	3	2	1	X
	**A quel point êtes-vous d'accord avec les affirmations suivantes ?**	**5 = très d'accord**	**4 = D'accord**	**3 = Neutre**	**2 = En desaccord**	**1 = Très en desaccord**	**ne sait pas/ ne dit rien**
Q68	Je dois effectuer des tâches inappropriées pendant mon stage	5	4	3	2	1	X
Q69	Il existe une discrimination basee sur le genre dans mes hopitaux de stages d'internat	5	4	3	2	1	X
Q70	Il existe d'autres formes de discrimination (par exemple, l'origine ethnique, la religion, la tribu, le handicap) dans mon hôpital de stage	5	4	3	2	1	X
Q71	Il y a une culture sans blâme dans mon hôpital d'internat	5	4	3	2	1	X
Q72	Je me en sécurité d'être traité comme un patient dans mon hôpital de stage	5	4	3	2	1	X
Q73	Les erreurs médicales sont traitees de manieres appropriée dans mon hopital de stage	5	4	3	2	1	X
Q74	Je connais les canaux appropriés pour adresser des questions concernant la sécurité des patients	5	4	3	2	1	X
Q75	Il est difficile de discuter des erreurs médicaux dans mon hopital de stage d'internat	5	4	3	2	1	X

Q76	Mes collègues m'encouragent à signaler tout problème de sécurité des patients que je pourrais avoir	5	4	3	2	1	X
Q77	La culture dans mon hôpital d'internat rend facile d'apprendre facilement des erreurs des autres	5	4	3	2	1	X
Q78	Je connais les canaux appropriés pour adresser des questions concernant ma propre sécurité	5	4	3	2	1	X
Q79	Je me sens émotionnellement vulnérable dans mon environnement hospitalier de stage	5	4	3	2	1	X
Q80	Je me sens physiquement en securité dans mon hospitalier de stage	5	4	3	2	1	X
Q81	Je suit intimidé ou victime dans mon hospital de stage	5	4	3	2	1	X
Q82	Ils existent des protocols de securité des patients clairs et à jour dans l'hôpital de stage.	5	4	3	2	1	X
Q83	Ils existent des mesures adéquates de prevention et de controle de l'infection	5	4	3	2	1	X
Q84	Je peux signaler toute préoccupation et recevoir des commentaires réactifs dans mon hôpital de stage.	5	4	3	2	1	X
Q85	L'hôpital de stage a un logement de bonne qualité pour moi lorsque je suis de garde	5	4	3	2	1	X
	**A quel point etes-vous d'accord avec les affirmations suivates?**	**5 = Très d'accord**	**4 = D'accord**	**3 = Neutre**	**2 = En desaccord**	**1 = très en desaccord**	**Ne sait pas/Ne dit rien**
Q86	Il existe des services de restauration adéquats fournis par l'hôpital de stage lorsque je suis de garde	5	4	3	2	1	X
Q87	L'hôpital de stage a une bonne connexion Internet pour mes besoins d'études et de travail	5	4	3	2	1	X
Q88	L'hôpital de stage dispose d'un approvisionnement suffisant en diagnostics, équipements et médicaments pour mes besoins d'études et de travail	5	4	3	2	1	X

这部分的问题将询问您在临床实习期间的感受和想法。同时也会询问您作为助人者时所经历的正面和负面经验。当您帮助他人时，会直接接接触到他人的生活。您或许也会发现您对所帮助对象的同情心也会对您产生正面和负面的影响。

请您综合思考所有临床科室轮转过程中的感受，而非您此时正在轮转的科室。
请您圈出您出现这种感受或想法的频率。

	在您的临床实习过程中，您出现以下感受或想法的频率为	5 = 总是如此	4 = 经常如此	3 = 有些时候	2 = 很少	1 = 从未有过	不清楚 / 不愿回答
Q1	我觉得在临床实习期间我无法平衡我的工作和个人生活。	5	4	3	2	1	X
Q2	我会因为实习工作感到紧张和压力。	5	4	3	2	1	X
Q3	在临床实习期间我相信我有能力解决我的个人问题。	5	4	3	2	1	X
Q4	我觉得我生命中的事情并没有按照我想要的方式发展。	5	4	3	2	1	X
Q5	我发现我不能很好地应对实习期间我需要完成的工作。	5	4	3	2	1	X
Q6	我有能力去控制实习期间因为工作和生活产生的烦躁情绪。	5	4	3	2	1	X
Q7	我觉得工作尽在我掌握之中。	5	4	3	2	1	X
Q8	我会因为事情超出我的控制而生气	5	4	3	2	1	X
Q9	我会因为工作堆积而感到无法克服的困难。	5	4	3	2	1	X
Q10	我对以前喜欢做的事情失去兴趣并无法从中获得快乐。	5	4	3	2	1	X
Q11	我因为实习工作感到低落、沮丧和绝望。	5	4	3	2	1	X
Q12	我有睡眠障碍，表现为入睡困难或难以保持睡眠状态，或是睡眠过多。	5	4	3	2	1	X
Q13	我在实习期间感到疲惫或能量不足。	5	4	3	2	1	X
Q14	我有进食障碍，表现为食欲不振或过度进食。	5	4	3	2	1	X
Q15	我对工作相关或是工作以外的事情难以集中注意力。	5	4	3	2	1	X
Q16	我曾想过死亡可能会让我好受一些，或者想过伤害自己。	5	4	3	2	1	X
Q17	我能从帮助他人的过程中获得满足感。	5	4	3	2	1	X
Q18	我在实习期间完成工作后感到精神饱满和充满能量。	5	4	3	2	1	X
Q19	我喜欢医学实习的工作。	5	4	3	2	1	X
Q20	能够跟上临床技能和流程的能力让我感到开心。	5	4	3	2	1	X
Q21	我的实习工作让我感到满足。	5	4	3	2	1	X

Q22	我相信我能做出有影响力的工作。	5	4	3	2	1	X
Q23	我为身为临床实习生所能做的工作感到自豪。	5	4	3	2	1	X
Q24	我相信我是一名成功的医学实习生。	5	4	3	2	1	X
Q25	我为我选择做这项工作而感到快乐。	5	4	3	2	1	X
Q26	我的实习经历与我的预期相符。	5	4	3	2	1	X
Q27	因为工作相关的创伤体验我出现睡眠不足的情况，这导致我无法像以往一样高效。	5	4	3	2	1	X
Q28	我觉得自己被医学实习工作所困。	5	4	3	2	1	X
Q29	我因为医学实习工作而感到疲惫。	5	4	3	2	1	X
Q30	我因为实习期间看似无穷无尽的工作感到不堪重负。	5	4	3	2	1	X
Q31	我感到被自己的实习医院所约束而停滞不前。	5	4	3	2	1	X
Q32	我在实习期间脑中会常常充满对于多个患者的担忧。	5	4	3	2	1	X
Q33	我难以将个人生活和医学实习生活区分开来。	5	4	3	2	1	X
Q34	我帮助的患者所经历的困难和压力会对我造成影响。	5	4	3	2	1	X
Q35	我因为帮助患者的工作而对许多事情感到紧张。	5	4	3	2	1	X
Q36	我会避免某些活动或场景，因为它们让我想到我的患者一些令人恐惧的经历。	5	4	3	2	1	X
Q37	因为我对患者的帮助，我会产生具有唐突、可怕的想法。	5	4	3	2	1	X

本环节将会询问您对目前所在的或之前所在的实习培训中心的感受和想法。同时也会询问您对您的临床导师（即在您实习期间为您提供监督和指导的人员）的感受和想法。
请圈出您对以下描述的认可程度。

	您对以下描述的认可程度为?	5 = 非常同意	4 = 同意	3 = 中立	2 = 不同意	1 = 非常不同意	不清楚 / 不愿回答
Q38	我的临床导师确立了明确的期望。	5	4	3	2	1	X

Q39	我在实习期间一直受到很好的临床指导。	5	4	3	2	1	X
Q40	实习期间医院安排分配了我自己阅读、训练、受教育的时间。	5	4	3	2	1	X
Q41	我的临床导师具有良好的沟通技巧	5	4	3	2	1	X
Q42	我在实习期间能积极参与教育课程（例如继续教育课程）。	5	4	3	2	1	X
Q43	我的临床导师对教学和督导充满热情。	5	4	3	2	1	X
Q44	我在实习期间能够接触到与我需求相关的教学课程和项目。	5	4	3	2	1	X
Q45	我的临床导师能定期给我反馈。	5	4	3	2	1	X
Q46	在实习期间我有足够满足我需求的临床学习机会。	5	4	3	2	1	X
Q47	在我需要时，我能获得临床导师的教学和督导。	5	4	3	2	1	X
Q48	我的临床导师鼓励我成为一个独立自主的学生。	5	4	3	2	1	X
Q49	我的临床导师会针对我的优势和劣势提供反馈以帮助我的专业发展。	5	4	3	2	1	X
Q50	我的临床导师具有良好的教学指导技巧。	5	4	3	2	1	X
Q51	我在实习期间有机会获得合适的临床实践操作流程。	5	4	3	2	1	X
Q52	我的实习训练让我觉得已经做好了成为独立临床执业者的准备。	5	4	3	2	1	X
Q53	我的临床导师提倡互相尊重的氛围。	5	4	3	2	1	X
Q54	我被行政管理工作占据而无法学习。	5	4	3	2	1	X
Q55	我参加过内容丰富、信息翔实的实习前入职介绍。	5	4	3	2	1	X
Q56	我有一本信息全面丰富的实习指南或临床手册。	5	4	3	2	1	X
Q57	作为一名医学实习生，我承担着恰当的工作责任。	5	4	3	2	1	X
Q58	我感到自己是团队的一部分。	5	4	3	2	1	X
Q59	实习医院的所有科室均有明确的临床流程和指南。	5	4	3	2	1	X
Q60	在实习期间我有机会为我的患者提供连续的医疗服务。	5	4	3	2	1	X

Q61	我能与其他临床执业者、实习生和医务人员进行良好的合作。	5	4	3	2	1	X
Q62	在实习期间我能获得职业生涯咨询服务，或者可以接触到职业生涯咨询顾问。	5	4	3	2	1	X
Q63	没有顺利如愿完成实习训练的医学实习生能获得咨询的机会。	5	4	3	2	1	X
Q64	在实习期间我和医院签署了关于工作时长的合同，或有说明了工作时长的其他文书材料。	5	4	3	2	1	X
Q65	实习期间我的工作时长是合适的。	5	4	3	2	1	X
Q66	实习期间我的工作量是合理的。	5	4	3	2	1	X
Q67	实习期间我会因为患者而被不恰当地呼叫或者联系。	5	4	3	2	1	X
Q68	实习期间我不得不做一些不恰当的工作。	5	4	3	2	1	X
Q69	我的实习医院存在性别歧视。	5	4	3	2	1	X
Q70	我的实习医院存在其他形式的歧视（例如种族、地域、残疾歧视）。	5	4	3	2	1	X
Q71	我的实习医院有“不责备”文化。	5	4	3	2	1	X
Q72	如果我自己在我的实习医院就诊，我会感到安全。	5	4	3	2	1	X
Q73	我的实习医院能够正确处理医疗差错。	5	4	3	2	1	X
Q74	我知道反映患者安全问题的正确渠道。	5	4	3	2	1	X
Q75	在我的实习医院很难讨论医疗差错。	5	4	3	2	1	X
Q76	我同事鼓励我去汇报任何关于患者安全的问题。	5	4	3	2	1	X
Q77	我所在实习医院的文化让我能够从别人的错误中学习。	5	4	3	2	1	X
Q78	我知道反映我个人安全问题的正确渠道。	5	4	3	2	1	X
Q79	我在实习医院的环境中觉得自己情绪脆弱。	5	4	3	2	1	X
Q80	我在实习医院中觉得人身安全得到了保障。	5	4	3	2	1	X
Q81	我在实习医院中受到欺凌或伤害。	5	4	3	2	1	X

Q82	我的实习医院有清晰且按时更新的关于患者安全问题的处理流程。	5	4	3	2	1	X
Q83	我的实习医院有充分的感染防控措施。	5	4	3	2	1	X
Q84	我在实习医院中能反映我的任何担忧并得到及时反馈。	5	4	3	2	1	X
Q85	我的实习医院能在值班时为我提供良好的住宿环境。	5	4	3	2	1	X
Q86	我的实习医院在值班时为我提供餐饮服务。	5	4	3	2	1	X
Q87	我的实习医院能提供良好的网络以满足我的学习和工作需求。	5	4	3	2	1	X
Q88	我的实习医院能提供充足的诊断工具，医疗设备及药品以满足我的学习和工作需求。	5	4	3	2	1	X

Những câu hỏi trong phần này sẽ hỏi bạn về những cảm xúc và suy nghĩ trong suốt những năm thực tập lâm sàng của bạn. Phần này cũng sẽ hỏi bạn về những trải nghiệm tích cực lẫn tiêu cực về sự căng thẳng, trầm cảm, kiệt sức hay sự hài lòng và các nội dung suy giảm sự đồng cảm ảnh hưởng đến bạn như thế nào trong quá trình thực tập.
Vui lòng phản hồi về toàn bộ trải nghiệm thực tập lâm sàng của bạn trong tất cả các lần luân chuyển giữa các khoa, chứ không chỉ về trải nghiệm với khoa đang thực tập hiện tại.
Vui lòng khoanh tròn mức độ thường xuyên mà bạn cảm thấy hoặc suy nghĩ theo cách bạn thường có.

STT	Trong năm thực tập của bạn, bạn thường trải qua những cảm giác hoặc suy nghĩ dưới đây ở mức độ nào?	5 = Rất thường xuyên	4 = Thường xuyên	3 = Thỉnh thoảng	2 = Hiếm khi	1 = Không bao giờ	Không biết/ Không muốn đề cập
	**Stress**						
Q1	Tôi cảm thấy không thể cân bằng giữa công việc và cuộc sống cá nhân trong thời gian thực tập.						
Q2	Tôi cảm thấy lo lắng hoặc căng thẳng vì công việc thực tập.						
Q3	Tôi cảm thấy tự tin về khả năng xử lý các vấn đề cá nhân trong thời gian thực tập.						

STT	Trong năm thực tập của bạn, bạn thường trải qua những cảm giác hoặc suy nghĩ dưới đây ở mức độ nào?	5 = Rất thường xuyên	4 = Thường xuyên	3 = Thỉnh thoảng	2 = Hiếm khi	1 = Không bao giờ	Không biết/ Không muốn đề cập
Q4	Tôi từng cảm thấy mọi thứ không đi theo hướng tôi mong đợi trong việc thực tập lẫn cuộc sống.						
Q5	Tôi nhận ra tôi không thể xử lý tất cả các công việc mà tôi phải làm trong thời gian thực tập.						
Q6	Tôi có thể kiểm soát những bực bội về công việc lẫn cuộc sống trong thời gian thực tập.						
Q7	Tôi cảm thấy tôi đang kiểm soát được mọi thứ trong công việc.						
Q8	Tôi cảm thấy khó chịu vì những thứ nằm ngoài tầm kiểm soát của tôi.						
Q9	Tôi cảm thấy những khó khăn trong công việc đang chồng chất đến mức mà tôi không thể vượt qua được.						
	**Trầm cảm**						
Q10	Tôi ít hứng thú hay ít hào hứng khi làm những việc mà trước kia tôi từng yêu thích.						
Q11	Tôi cảm thấy xuống tinh thần, chán nản hoặc tuyệt vọng vì công việc thực tập của mình.						
Q12	Tôi bị rối loạn giấc ngủ, khó ngủ, ngủ chập chờn hoặc ngủ quá nhiều.						
Q13	Tôi cảm thấy mệt mỏi hoặc có ít năng lượng trong thời gian thực tập.						
Q14	Tôi có vấn đề về ăn uống, như chán ăn hoặc ăn quá nhiều.						

STT	Trong năm thực tập của bạn, bạn thường trải qua những cảm giác hoặc suy nghĩ dưới đây ở mức độ nào?	5 = Rất thường xuyên	4 = Thường xuyên	3 = Thỉnh thoảng	2 = Hiếm khi	1 = Không bao giờ	Không biết/ Không muốn đề cập
Q15	Tôi khó tập trung vào những thứ cả trong và ngoài công việc.						
Q16	Tôi có ý định tự tử hoặc làm tổn thương bản thân bằng một cách nào đó.						
	**Hài lòng**						
Q17	Tôi cảm thấy thỏa mãn khi có thể giúp đỡ mọi người.						
Q18	Công việc thực tập khiến tôi cảm thấy như được tiếp thêm sinh lực sau giờ làm việc						
Q19	Tôi thích công việc bác sĩ thực tập của mình.						
Q20	Khả năng theo kịp các kỹ thuật lâm sàng và phác đồ điều trị khiến tôi cảm thấy hài lòng.						
Q21	Công việc thực tập khiến tôi cảm thấy hài lòng.						
Q22	Tôi tin rằng công việc thực tập của tôi góp phần tạo nên sự khác biệt trong việc chăm sóc người bệnh.						
Q23	Tôi tự hào về những gì tôi có thể làm để giúp ích với tư cách là bác sĩ thực tập.						
Q24	Tôi tin rằng tôi thành công với tư cách là một bác sĩ thực tập.						
Q25	Tôi cảm thấy hạnh phúc, thoải mái khi làm việc ở vị trí bác sĩ thực tập.						
Q26	Trải nghiệm trong kỳ thực tập đáp ứng được kì vọng của tôi.						
	**Kiệt sức**						
Q27	Tôi làm việc không hiệu quả do mất ngủ bởi những sang chấn liên quan đến công việc						

STT	Trong năm thực tập của bạn, bạn thường trải qua những cảm giác hoặc suy nghĩ dưới đây ở mức độ nào?	5 = Rất thường xuyên	4 = Thường xuyên	3 = Thỉnh thoảng	2 = Hiếm khi	1 = Không bao giờ	Không biết/ Không muốn đề cập
Q28	Tôi cảm thấy bị mắc kẹt cả thể chất lẫn tinh thần trong khi thực tập						
Q29	Tôi cảm thấy bị hao mòn về thể chất và tinh thần trong quá trình thực tập						
Q30	Tôi cảm thấy quá tải vì khối lượng công việc dường như không bao giờ kết thúc						
Q31	Tôi cảm thấy bị giới hạn bởi bệnh viện nơi tôi thực tập.						
	**Suy giảm sự đồng cảm**						
Q32	Tôi phải bận tâm với nhiều người bệnh mà tôi điều trị và giúp đỡ trong quá trình thực tập.						
Q33	Tôi cảm thấy khó khăn để tách biệt cuộc sống cá nhân với cuộc sống của một bác sĩ thực tập.						
Q34	Tôi đã bị ảnh hưởng bởi sự khó khăn và căng thẳng của những người bệnh mà tôi điều trị và giúp đỡ.						
Q35	Tôi cảm thấy căng thẳng về nhiều thứ khác nhau vì việc giúp đỡ người bệnh.						
Q36	Tôi tránh một số hoạt động nhất định khi giúp đỡ người bệnh vì gợi nhớ về những trải nghiệm tồi tệ của những bệnh nhân mà tôi giúp đỡ trước đây.						
Q37	Việc giúp đỡ cho người bệnh khiến tôi có những ám ảnh và sợ hãi.						

Suy nghĩ hay góc nhìn của bạn về bệnh viện mà bạn hiện đang thực tập hoặc đã thực tập trước đó. Và cũng hỏi nhận thức của bạn về những người hướng dẫn, tức là những người đã giảng dạy và giám sát cho bạn trong thời gian thực tập.
Hãy khoanh tròn mức độ bạn đồng ý với những nhận định này.

STT	Bạn đồng ý với những nhận định sau ở mức độ nào?	5 = Rất đồng ý	4 = Đồng ý	3 = Trung tính	2 = Không đồng ý	1 = Rất không đồng ý	Không biết/ Không muốn đề cập
	**Giảng dạy và giám sát**						
Q38	Những bác sĩ hướng dẫn lâm sàng của tôi đặt ra những mục tiêu rõ ràng.						
Q39	Tôi luôn được hướng dẫn lâm sàng kĩ càng trong suốt quá trình thực tập						
Q40	Thời gian dành riêng cho việc tự học của bác sĩ thực tập được bệnh viện phân bổ rõ ràng.						
Q41	Những bác sĩ hướng dẫn lâm sàng của tôi có kỹ năng giao tiếp tốt.						
Q42	Tôi có thể tham gia tích cực vào các buổi đào tạo (Ví dụ như chương trình đào tạo y khoa liên tục – CME) trong quá trình thực tập.						
Q43	Những bác sĩ hướng dẫn lâm sàng của tôi rất nhiệt tình giảng dạy và hướng dẫn.						
Q44	Tôi được tham gia vào các chương trình học phù hợp với nhu cầu trong quá trình thực tập.						
Q45	Những bác sĩ hướng dẫn lâm sàng chủ động phản hồi thường xuyên cho tôi.						
Q46	Tôi có đủ cơ hội học tập để đáp ứng những nhu cầu về mặt chuyên môn trong suốt giai đoạn thực tập.						
Q47	Tôi thường dễ dàng tiếp cận những bác sĩ hướng dẫn lâm sàng để được giảng dạy và hướng dẫn						

STT	Bạn đồng ý với những nhận định sau ở mức độ nào?	5 = Rất đồng ý	4 = Đồn g ý	3 = Trung tính	2 = Khôn g đồng ý	1 = Rất không đồng ý	Không biết/ Không muốn đề cập
Q48	Những bác sĩ hướng dẫn lâm sàng khuyến khích tôi trở thành một người học độc lập.						
Q49	Những bác sĩ hướng dẫn phản hồi cho tôi về những điểm mạnh và điểm yếu của mình nhằm giúp tôi phát triển sự nghiệp						
Q50	Những bác sĩ hướng dẫn lâm sàng của tôi có các kỹ năng cố vấn tốt.						
Q51	Tôi có cơ hội tiếp thu các quy trình thực hành thích hợp cho việc thực hành lâm sàng trong quá trình thực tập của mình						
Q52	Chương trình thực tập của tôi giúp tôi cảm thấy sẵn sàng trở thành một bác sĩ thực hành độc lập.						
Q53	Những bác sĩ hướng dẫn lâm sàng của tôi khuyến khích bầu không khí làm việc có sự tôn trọng lẫn nhau.						
Q54	Sự bận rộn với công việc hành chính đã cản trở khả năng học hỏi của tôi.						
	**Hỗ trợ tổ chức**						
Q55	Tôi được tham gia một chương trình giới thiệu cho khoá thực tập với nhiều thông tin hữu ích tại bệnh viện thực tập.						
Q56	Tại các khoa có đầy đủ các hướng dẫn thực tập, sổ ghi chép hoặc nhật ký lâm sàng.						
Q57	Tôi cảm thấy mình làm tròn trách nhiệm với tư cách là một bác sĩ thực tập.						
Q58	Tôi cảm thấy mình là một phần của một nhóm làm việc ở đây.						
Q59	Có các hướng dẫn và phác đồ điều trị rõ ràng tại tất cả các khoa ở bệnh viện thực tập.						

STT	Bạn đồng ý với những nhận định sau ở mức độ nào?	5 = Rất đồng ý	4 = Đồn g ý	3 = Trung tính	2 = Khôn g đồng ý	1 = Rất không đồng ý	Không biết/ Không muốn đề cập
Q60	Tôi có cơ hội theo dõi điều trị liên tục cho người bệnh trong thời gian thực tập của mình.						
Q61	Tôi phối hợp tốt với các bác sĩ chính thức, bác sĩ thực tập và nhân viên y tế khác.						
Q62	Tôi có thể tiếp cận với các dịch vụ tư vấn nghề nghiệp phù hợp trong quá trình thực tập						
Q63	Có cơ hội để những thực tập sinh không đạt yêu cầu trong khóa đào tạo được tư vấn hỗ trợ tại bệnh viện thực tập.						
	**Điều kiện làm việc và an toàn**						
Q64	Tôi có hợp đồng lao động hoặc các văn bản khác cung cấp thông tin về số giờ làm việc trong thời gian thực tập.						
Q65	Số giờ làm việc của tôi trong thời gian thực tập là phù hợp.						
Q66	Khối lượng công việc của tôi trong thời gian thực tập là hợp lý.						
Q67	Tôi thường xuyên bị gọi vào bệnh viện vì những vấn đề liên quan đến bệnh nhân một cách không phù hợp trong quá trình thực tập.						
Q68	Tôi phải thực hiện các nhiệm vụ không phù hợp trong quá trình thực tập.						
Q69	Bệnh viện tôi thực tập có sự phân biệt về giới tính.						
Q70	Bệnh viện tôi thực tập có những hình thức phân biệt đối xử khác (ví dụ: sắc tộc, tôn giáo, dân tộc, khuyết tật).						
Q71	Bệnh viện tôi thực tập có văn hóa không đổ lỗi.						
Q72	Tôi sẽ cảm thấy an toàn khi được đối xử như một người bệnh trong bệnh viện mà tôi thực tập.						

STT	Bạn đồng ý với những nhận định sau ở mức độ nào?	5 = Rất đồng ý	4 = Đồng ý	3 = Trung tính	2 = Không đồng ý	1 = Rất không đồng ý	Không biết/ Không muốn đề cập
Q73	Các sai sót y khoa được bệnh viện xử lý thích hợp trong bệnh viện mà tôi thực tập.						
Q74	Tôi biết các trang web, nguồn tài liệu để tìm thông tin liên quan đến sự an toàn người bệnh tại bệnh viện thực tập.						
Q75	Tại bệnh viện tôi thực tập rất khó để thảo luận về những sai sót.						
Q76	Tôi được các đồng nghiệp khuyến khích báo cáo bất kỳ mối lo ngại nào về an toàn người bệnh mà tôi có thể có.						
Q77	Văn hóa ở bệnh viện tôi thực tập giúp tôi dễ dàng học hỏi từ những sai sót của người khác.						
Q78	Tôi biết các kênh thích hợp để trả lời các câu hỏi trực tiếp liên quan đến sự an toàn của chính bản thân tôi.						
Q79	Tôi cảm thấy dễ bị tổn thương về mặt cảm xúc trong môi trường bệnh viện tôi thực tập.						
Q80	Tôi cảm thấy an toàn về thể chất trong bệnh viện nơi tôi thực tập.						
Q81	Tôi bị bắt nạt hoặc trở thành nạn nhân trong bệnh viện nơi tôi thực tập.						
Q82	Bệnh viện thực tập có các quy trình an toàn người bệnh rõ ràng và cập nhật.						
Q83	Có đầy đủ các biện pháp phòng ngừa kiểm soát nhiễm khuẩn.						
Q84	Tôi có thể báo cáo bất kỳ sự lo ngại nào và nhận được phản hồi nhanh chóng tại bệnh viện thực tập.						
	**Nguồn lực vật chất và tài chính**						
Q85	Bệnh viện thực tập có chỗ ở với chất lượng tốt khi tôi trực.						

STT	Bạn đồng ý với những nhận định sau ở mức độ nào?	5 = Rất đồng ý	4 = Đồng ý	3 = Trung tính	2 = Không đồng ý	1 = Rất không đồng ý	Không biết/ Không muốn đề cập
Q86	Có đầy đủ các dịch vụ ăn uống do bệnh viện thực tập cung cấp khi tôi trực.						
Q87	Bệnh viện thực tập có đường truyền internet tốt phục vụ nhu cầu học tập và làm việc.						
Q88	Bệnh viện cung cấp đầy đủ thiết bị chẩn đoán, thuốc men phục vụ nhu cầu học tập và làm việc.						

Supplementary appendix 5. Missing values and inter-total correlations

Table a. Missing value by items and in countries with over 10 responses

Country	**Total**	Uganda	Kenya	Vietnam	China	Burundi	Sierra Leone	Nigeria	Fiji	South Africa	Tanzania	Ghana
Sample size	**1646**	487	358	177	160	120	98	90	42	39	25	17
q1_pc	**0.4%**	0.0%	0.0%	0.0%	0.0%	0.0%	0.0%	0.0%	0.0%	0.0%	0.0%	0.0%
q2_pc	**0.1%**	0.0%	0.0%	0.0%	0.0%	0.0%	0.0%	0.0%	0.0%	0.0%	0.0%	0.0%
q3_pc	**0.5%**	0.2%	0.3%	0.6%	0.6%	0.8%	1.0%	1.1%	2.4%	2.6%	4.0%	5.9%
q4_pc	**1.3%**	0.2%	0.3%	0.6%	0.6%	0.8%	1.0%	1.1%	2.4%	2.6%	4.0%	5.9%
q5_pc	**0.7%**	0.0%	0.0%	0.0%	0.0%	0.0%	0.0%	0.0%	0.0%	0.0%	0.0%	0.0%
q6_pc	**0.4%**	0.2%	0.3%	0.6%	0.6%	0.8%	1.0%	1.1%	2.4%	2.6%	4.0%	5.9%
q7_pc	**0.5%**	0.0%	0.0%	0.0%	0.0%	0.0%	0.0%	0.0%	0.0%	0.0%	0.0%	0.0%
q8_pc	**0.4%**	0.2%	0.3%	0.6%	0.6%	0.8%	1.0%	1.1%	2.4%	2.6%	4.0%	5.9%
q9_pc	**0.4%**	0.2%	0.3%	0.6%	0.6%	0.8%	1.0%	1.1%	2.4%	2.6%	4.0%	5.9%
q10_pc	**0.7%**	0.0%	0.0%	0.0%	0.0%	0.0%	0.0%	0.0%	0.0%	0.0%	0.0%	0.0%
q11_pc	**0.6%**	0.2%	0.3%	0.6%	0.6%	0.8%	1.0%	1.1%	2.4%	2.6%	4.0%	5.9%
q12_pc	**0.4%**	0.0%	0.0%	0.0%	0.0%	0.0%	0.0%	0.0%	0.0%	0.0%	0.0%	0.0%
q13_pc	**0.1%**	0.2%	0.3%	0.6%	0.6%	0.8%	1.0%	1.1%	2.4%	2.6%	4.0%	5.9%
q14_pc	**0.5%**	0.2%	0.3%	0.6%	0.6%	0.8%	1.0%	1.1%	2.4%	2.6%	4.0%	5.9%
q15_pc	**0.2%**	0.2%	0.3%	0.6%	0.6%	0.8%	1.0%	1.1%	2.4%	2.6%	4.0%	5.9%
q16_pc	**1.2%**	0.6%	0.8%	1.7%	1.9%	2.5%	3.1%	3.2%	7.1%	7.7%	12.0%	17.6%
q17_pc	**0.1%**	0.0%	0.0%	0.0%	0.0%	0.0%	0.0%	0.0%	0.0%	0.0%	0.0%	0.0%
q18_pc	**0.5%**	0.2%	0.3%	0.6%	0.6%	0.8%	1.0%	1.1%	2.4%	2.6%	4.0%	5.9%
q19_pc	**0.8%**	0.4%	0.6%	1.1%	1.3%	1.7%	2.0%	2.2%	4.8%	5.1%	8.0%	11.8%
q20_pc	**0.5%**	0.2%	0.3%	0.6%	0.6%	0.8%	1.0%	1.1%	2.4%	2.6%	4.0%	5.9%
q21_pc	**0.1%**	0.0%	0.0%	0.0%	0.0%	0.0%	0.0%	0.0%	0.0%	0.0%	0.0%	0.0%

q22_pc	**0.5%**	0.2%	0.3%	0.6%	0.6%	0.8%	1.0%	1.1%	2.4%	2.6%	4.0%	5.9%
q23_pc	**0.2%**	0.0%	0.0%	0.0%	0.0%	0.0%	0.0%	0.0%	0.0%	0.0%	0.0%	0.0%
q24_pc	**1.3%**	0.4%	0.6%	1.1%	1.3%	1.7%	2.0%	2.2%	4.8%	5.1%	8.0%	11.8%
q25_pc	**0.4%**	0.4%	0.6%	1.1%	1.3%	1.7%	2.0%	2.2%	4.8%	5.1%	8.0%	11.8%
q26_pc	**1.4%**	0.4%	0.6%	1.1%	1.3%	1.7%	2.0%	2.2%	4.8%	5.1%	8.0%	11.8%
q27_pc	**0.5%**	0.0%	0.0%	0.0%	0.0%	0.0%	0.0%	0.0%	0.0%	0.0%	0.0%	0.0%
q28_pc	**0.6%**	0.2%	0.3%	0.6%	0.6%	0.8%	1.0%	1.1%	2.4%	2.6%	4.0%	5.9%
q29_pc	**0.3%**	0.0%	0.0%	0.0%	0.0%	0.0%	0.0%	0.0%	0.0%	0.0%	0.0%	0.0%
q30_pc	**0.2%**	0.2%	0.3%	0.6%	0.6%	0.8%	1.0%	1.1%	2.4%	2.6%	4.0%	5.9%
q31_pc	**1.0%**	0.2%	0.3%	0.6%	0.6%	0.8%	1.0%	1.1%	2.4%	2.6%	4.0%	5.9%
q32_pc	**0.5%**	0.0%	0.0%	0.0%	0.0%	0.0%	0.0%	0.0%	0.0%	0.0%	0.0%	0.0%
q33_pc	**0.7%**	0.0%	0.0%	0.0%	0.0%	0.0%	0.0%	0.0%	0.0%	0.0%	0.0%	0.0%
q34_pc	**0.5%**	0.2%	0.3%	0.6%	0.6%	0.8%	1.0%	1.1%	2.4%	2.6%	4.0%	5.9%
q35_pc	**1.2%**	0.0%	0.0%	0.0%	0.0%	0.0%	0.0%	0.0%	0.0%	0.0%	0.0%	0.0%
q36_pc	**1.0%**	0.6%	0.8%	1.7%	1.9%	2.5%	3.1%	3.2%	7.1%	7.7%	12.0%	17.6%
q37_pc	**1.2%**	0.2%	0.3%	0.6%	0.6%	0.8%	1.0%	1.1%	2.4%	2.6%	4.0%	5.9%
q38_pc	**0.5%**	0.0%	0.0%	0.0%	0.0%	0.0%	0.0%	0.0%	0.0%	0.0%	0.0%	0.0%
q39_pc	**1.7%**	0.0%	0.0%	0.0%	0.0%	0.0%	0.0%	0.0%	0.0%	0.0%	0.0%	0.0%
q40_pc	**0.9%**	0.2%	0.3%	0.6%	0.6%	0.8%	1.0%	1.1%	2.4%	2.6%	4.0%	5.9%
q41_pc	**0.5%**	0.0%	0.0%	0.0%	0.0%	0.0%	0.0%	0.0%	0.0%	0.0%	0.0%	0.0%
q42_pc	**0.7%**	0.0%	0.0%	0.0%	0.0%	0.0%	0.0%	0.0%	0.0%	0.0%	0.0%	0.0%
q43_pc	**0.7%**	0.0%	0.0%	0.0%	0.0%	0.0%	0.0%	0.0%	0.0%	0.0%	0.0%	0.0%
q44_pc	**0.5%**	0.0%	0.0%	0.0%	0.0%	0.0%	0.0%	0.0%	0.0%	0.0%	0.0%	0.0%
q45_pc	**0.7%**	0.0%	0.0%	0.0%	0.0%	0.0%	0.0%	0.0%	0.0%	0.0%	0.0%	0.0%
q46_pc	**0.4%**	0.0%	0.0%	0.0%	0.0%	0.0%	0.0%	0.0%	0.0%	0.0%	0.0%	0.0%
q47_pc	**0.7%**	0.0%	0.0%	0.0%	0.0%	0.0%	0.0%	0.0%	0.0%	0.0%	0.0%	0.0%
q48_pc	**1.2%**	0.0%	0.0%	0.0%	0.0%	0.0%	0.0%	0.0%	0.0%	0.0%	0.0%	0.0%
q49_pc	**1.4%**	0.0%	0.0%	0.0%	0.0%	0.0%	0.0%	0.0%	0.0%	0.0%	0.0%	0.0%
q50_pc	**1.0%**	0.2%	0.3%	0.6%	0.6%	0.8%	1.0%	1.1%	2.4%	2.6%	4.0%	5.9%
q51_pc	**1.3%**	0.0%	0.0%	0.0%	0.0%	0.0%	0.0%	0.0%	0.0%	0.0%	0.0%	0.0%
q52_pc	**1.0%**	0.0%	0.0%	0.0%	0.0%	0.0%	0.0%	0.0%	0.0%	0.0%	0.0%	0.0%
q53_pc	**1.6%**	0.0%	0.0%	0.0%	0.0%	0.0%	0.0%	0.0%	0.0%	0.0%	0.0%	0.0%
q54_pc	**1.0%**	0.0%	0.0%	0.0%	0.0%	0.0%	0.0%	0.0%	0.0%	0.0%	0.0%	0.0%
q55_pc	**1.1%**	0.4%	0.6%	1.1%	1.3%	1.7%	2.0%	2.2%	4.8%	5.1%	8.0%	11.8%
q56_pc	**1.0%**	0.2%	0.3%	0.6%	0.6%	0.8%	1.0%	1.1%	2.4%	2.6%	4.0%	5.9%
q57_pc	**0.9%**	0.0%	0.0%	0.0%	0.0%	0.0%	0.0%	0.0%	0.0%	0.0%	0.0%	0.0%
q58_pc	**1.5%**	0.0%	0.0%	0.0%	0.0%	0.0%	0.0%	0.0%	0.0%	0.0%	0.0%	0.0%
q59_pc	**0.8%**	0.2%	0.3%	0.6%	0.6%	0.8%	1.0%	1.1%	2.4%	2.6%	4.0%	5.9%
q60_pc	**0.1%**	0.0%	0.0%	0.0%	0.0%	0.0%	0.0%	0.0%	0.0%	0.0%	0.0%	0.0%
q61_pc	**0.2%**	0.0%	0.0%	0.0%	0.0%	0.0%	0.0%	0.0%	0.0%	0.0%	0.0%	0.0%
q62_pc	**0.5%**	0.2%	0.3%	0.6%	0.6%	0.8%	1.0%	1.1%	2.4%	2.6%	4.0%	5.9%
q63_pc	**4.6%**	0.8%	1.1%	2.3%	2.5%	3.3%	4.1%	4.3%	9.5%	10.3%	16.0%	23.5%
q64_pc	**1.2%**	0.2%	0.3%	0.6%	0.6%	0.8%	1.0%	1.1%	2.4%	2.6%	4.0%	5.9%
q65_pc	**0.4%**	0.2%	0.3%	0.6%	0.6%	0.8%	1.0%	1.1%	2.4%	2.6%	4.0%	5.9%
q66_pc	**0.4%**	0.0%	0.0%	0.0%	0.0%	0.0%	0.0%	0.0%	0.0%	0.0%	0.0%	0.0%
q67_pc	**1.4%**	0.4%	0.6%	1.1%	1.3%	1.7%	2.0%	2.2%	4.8%	5.1%	8.0%	11.8%
q68_pc	**0.7%**	0.2%	0.3%	0.6%	0.6%	0.8%	1.0%	1.1%	2.4%	2.6%	4.0%	5.9%
q69_pc	**1.2%**	0.4%	0.6%	1.1%	1.3%	1.7%	2.0%	2.2%	4.8%	5.1%	8.0%	11.8%

q70_pc	**1.5%**	0.4%	0.6%	1.1%	1.3%	1.7%	2.0%	2.2%	4.8%	5.1%	8.0%	11.8%
q71_pc	**2.9%**	0.0%	0.0%	0.0%	0.0%	0.0%	0.0%	0.0%	0.0%	0.0%	0.0%	0.0%
q72_pc	**1.2%**	0.4%	0.6%	1.1%	1.3%	1.7%	2.0%	2.2%	4.8%	5.1%	8.0%	11.8%
q73_pc	**1.7%**	0.4%	0.6%	1.1%	1.3%	1.7%	2.0%	2.2%	4.8%	5.1%	8.0%	11.8%
q74_pc	**2.0%**	0.2%	0.3%	0.6%	0.6%	0.8%	1.0%	1.1%	2.4%	2.6%	4.0%	5.9%
q75_pc	**1.6%**	1.2%	1.7%	3.4%	3.8%	5.0%	6.1%	6.5%	14.3%	15.4%	24.0%	35.3%
q76_pc	**1.9%**	0.2%	0.3%	0.6%	0.6%	0.8%	1.0%	1.1%	2.4%	2.6%	4.0%	5.9%
q77_pc	**0.9%**	0.0%	0.0%	0.0%	0.0%	0.0%	0.0%	0.0%	0.0%	0.0%	0.0%	0.0%
q78_pc	**1.8%**	0.2%	0.3%	0.6%	0.6%	0.8%	1.0%	1.1%	2.4%	2.6%	4.0%	5.9%
q79_pc	**1.1%**	0.0%	0.0%	0.0%	0.0%	0.0%	0.0%	0.0%	0.0%	0.0%	0.0%	0.0%
q80_pc	**0.5%**	0.0%	0.0%	0.0%	0.0%	0.0%	0.0%	0.0%	0.0%	0.0%	0.0%	0.0%
q81_pc	**0.9%**	0.6%	0.8%	1.7%	1.9%	2.5%	3.1%	3.2%	7.1%	7.7%	12.0%	17.6%
q82_pc	**2.8%**	0.4%	0.6%	1.1%	1.3%	1.7%	2.0%	2.2%	4.8%	5.1%	8.0%	11.8%
q83_pc	**0.4%**	0.0%	0.0%	0.0%	0.0%	0.0%	0.0%	0.0%	0.0%	0.0%	0.0%	0.0%
q84_pc	**1.2%**	0.4%	0.6%	1.1%	1.3%	1.7%	2.0%	2.2%	4.8%	5.1%	8.0%	11.8%
q85_pc	**0.6%**	0.2%	0.3%	0.6%	0.6%	0.8%	1.0%	1.1%	2.4%	2.6%	4.0%	5.9%
q86_pc	**0.4%**	0.0%	0.0%	0.0%	0.0%	0.0%	0.0%	0.0%	0.0%	0.0%	0.0%	0.0%
q87_pc	**0.4%**	0.0%	0.0%	0.0%	0.0%	0.0%	0.0%	0.0%	0.0%	0.0%	0.0%	0.0%
q88_pc	**0.6%**	0.0%	0.0%	0.0%	0.0%	0.0%	0.0%	0.0%	0.0%	0.0%	0.0%	0.0%

Table b. Inter-item and item-total correlations for MIES items

Item	Item-test correlation	Item-rest correlation	Average interitem covariance	Alpha
Q1	0.4581	0.4383	0.2997761	0.9658
Q2	0.5888	0.573	0.2980044	0.9655
Q3	0.3945	0.3772	0.3017657	0.9659
Q4	0.4965	0.4789	0.299638	0.9657
Q5	0.5382	0.5222	0.2992985	0.9656
Q6	0.3106	0.2923	0.3028848	0.966
Q7	0.3711	0.3539	0.3021822	0.9659
Q8	0.463	0.4446	0.3001186	0.9657
Q9	0.5529	0.5377	0.2993012	0.9655
Q10	0.466	0.4467	0.2997372	0.9657
Q11	0.6345	0.6198	0.2972076	0.9654
Q12	0.5051	0.485	0.2984925	0.9657
Q13	0.5861	0.5705	0.2982011	0.9655
Q14	0.5143	0.4938	0.2980819	0.9657
Q15	0.5436	0.5276	0.2991956	0.9656
Q16	0.3917	0.3748	0.301897	0.9659
Q17	0.2793	0.263	0.3036898	0.966
Q18	0.4667	0.4484	0.3000484	0.9657
Q19	0.5915	0.577	0.2986353	0.9655
Q20	0.4652	0.45	0.3011341	0.9657
Q21	0.5868	0.5735	0.299367	0.9655
Q22	0.42	0.4034	0.3015109	0.9658
Q23	0.4092	0.3926	0.3016959	0.9658
Q24	0.4372	0.4202	0.3011026	0.9658
Q25	0.5594	0.5443	0.2991695	0.9655

Q26	0.6231	0.6089	0.2978709	0.9654
Q27	0.4807	0.4632	0.3000374	0.9657
Q28	0.5724	0.5551	0.2977941	0.9655
Q29	0.5806	0.5642	0.2979863	0.9655
Q30	0.5695	0.5529	0.2981939	0.9655
Q31	0.6396	0.6246	0.296814	0.9653
Q32	0.3747	0.3551	0.301533	0.9659
Q33	0.4713	0.4515	0.2994757	0.9657
Q34	0.411	0.3919	0.3009653	0.9658
Q35	0.4451	0.4277	0.3008283	0.9658
Q36	0.3738	0.3542	0.3015335	0.9659
Q37	0.4064	0.3882	0.3013053	0.9658
Q38	0.5605	0.5457	0.2992725	0.9655
Q39	0.6372	0.6234	0.2976769	0.9654
Q40	0.5324	0.5148	0.2987565	0.9656
Q41	0.6418	0.6289	0.2980672	0.9654
Q42	0.5459	0.5302	0.2992768	0.9656
Q43	0.6169	0.6034	0.2985037	0.9654
Q44	0.6336	0.6201	0.2979651	0.9654
Q45	0.6068	0.5924	0.2982355	0.9654
Q46	0.5464	0.5315	0.2995764	0.9656
Q47	0.6216	0.6089	0.298781	0.9654
Q48	0.4166	0.4014	0.3019687	0.9658
Q49	0.6051	0.5911	0.2985078	0.9654
Q50	0.6768	0.665	0.2976588	0.9653
Q51	0.4769	0.4624	0.3011753	0.9657
Q52	0.4886	0.4733	0.3006858	0.9657
Q53	0.6612	0.6488	0.2977257	0.9653
Q54	0.1407	0.1182	0.3048978	0.9664
Q55	0.4192	0.4003	0.3008605	0.9658
Q56	0.2942	0.2717	0.3024003	0.9661
Q57	0.5323	0.5166	0.2995299	0.9656
Q58	0.5906	0.5772	0.2992044	0.9655
Q59	0.6173	0.6026	0.2977834	0.9654
Q60	0.5096	0.4951	0.3005707	0.9656
Q61	0.5164	0.5033	0.3010109	0.9656
Q62	0.6002	0.5844	0.2976888	0.9654
Q63	0.591	0.5743	0.2975467	0.9654
Q64	0.4065	0.3836	0.3000663	0.9659
Q65	0.5735	0.5556	0.2974395	0.9655
Q66	0.5779	0.5606	0.2975927	0.9655
Q67	0.3203	0.2986	0.3020851	0.9661
Q68	0.4417	0.4214	0.299982	0.9658
Q69	0.4212	0.4019	0.3007181	0.9658
Q70	0.4146	0.3943	0.3005865	0.9659
Q71	0.2957	0.2742	0.3025409	0.9661
Q72	0.4934	0.4747	0.2992839	0.9657
Q73	0.5864	0.5718	0.2987136	0.9655
Q74	0.5643	0.5496	0.2992657	0.9655
Q75	0.4684	0.451	0.300353	0.9657

Q76	0.5081	0.4929	0.3002988	0.9656
Q77	0.5921	0.5786	0.2991131	0.9655
Q78	0.5973	0.583	0.2985506	0.9655
Q79	0.493	0.4758	0.2998456	0.9657
Q80	0.4911	0.4756	0.3005158	0.9657
Q81	0.5003	0.4827	0.2995547	0.9657
Q82	0.5742	0.5594	0.2989493	0.9655
Q83	0.5443	0.5277	0.2988777	0.9656
Q84	0.6629	0.6502	0.2974477	0.9653
Q85	0.4601	0.438	0.2989835	0.9658
Q86	0.4332	0.4107	0.2995018	0.9659
Q87	0.4395	0.4166	0.2992504	0.9659
Q88	0.5098	0.4901	0.2984895	0.9657
Test scale			0.2996569	0.966

Supplementary appendix 6. Three-factor structure for MIES analysis

Factor	Item no.	Question	Item loading	Original scale
Factor 1 – Educational and work environment (n=33) (Cronbach's alpha 0.95)	q39	I have good clinical supervision at all times during my internship.	0.6171	Adapted from Postgraduate Hospital Educational Environment Measure (PHEEM)
	q40	Time for my own reading, training and education is allocated by the hospital during my internship.	0.557	
	q41	My clinical supervisors have good communication skills.	0.5709	
	q42	I am able to participate actively in educational sessions (e.g. continuing medical educations) during my internship.	0.4976	
	q43	My clinical supervisors are enthusiastic about teaching and supervision.	0.6251	
	q44	I have access to educational sessions and programmes that are relevant to my needs during my internship	0.6603	
	q45	The clinical supervisors provide me with regular feedback.	0.6818	
	q46	I have enough clinical learning opportunities for my needs during the internship period.	0.5441	
	q47	My clinical supervisors are accessible for teaching and supervision.	0.6427	
	q49	The clinical supervisors provide me with feedback on my strengths and weaknesses to ensure my professional development.	0.6579	
	q50	My clinical supervisors have good mentoring skills.	0.6677	
	q53	My clinical supervisors promote an atmosphere of mutual respect.	0.5353	
	q55	I had an informative internship induction programme.	0.5152	
	q57	I have the appropriate level of responsibility as a medical intern.	0.4929	
	q59	There are clear clinical protocols and guidelines across all departments in the internship hospital.	0.6773	
	q62	I have suitable access to careers advice services or advisors during my internship.	0.6419	
	q63	There are good counselling opportunities for medical interns who fail to complete their training satisfactorily.	0.6572	
	q64	I have a contract of employment or other document that provides information about hours of work during my internship.	0.536	
	q65	My work hours are appropriate during my internship.	0.5787	
	q72	I would feel safe being treated as a patient in my internship hospital.	0.5524	Adapted from Safety Attitude Questionnaire
	q73	Medical errors are handled appropriately in my internship hospital.	0.6359	
	q74	I know the proper channels to direct questions regarding patient safety.	0.6034	
	q76	I am encouraged by my colleagues to report any patient safety concerns I may have.	0.5662	
	q77	The culture in my internship hospital makes it easy to learn from the errors of others.	0.6047	
	q78	I know the proper channels to direct questions regarding my own safety.	0.5966	New

	q80	I feel physically safe within my internship hospital.	0.3672	Adapted from PHEEM
	q82	There are clear and updated patient safety protocols in the internship hospital.	0.6771	New
	q83	There are adequate infection prevention and control measures.	0.6249	New
	q84	I can report any concern and receive responsive feedback in my internship hospital.	0.7385	Adapted from PHEEM
	q85	The internship hospital has good quality accommodation for me when on call.	0.6083	
	q86	There are adequate catering services provided by the internship hospital when I am on call.	0.6232	
	q87	The internship hospital has good internet connection for my study and work need.	0.5691	New
	q88	The internship hospital has adequate supply of diagnostics, equipment and medication for my study and work need.	0.6605	New
Factor 2 – Wellbeing (n=24) (Cronbach's alpha 0.94)	q1	I feel that I am unable to balance my work and personal life during my internship.	0.63	Adapted from Perceived Stress Scale
	q2	I feel nervous and/or stressed because of my internship work.	0.6581	
	q4	I have felt that things have not gone my way in my life.	0.4961	
	q5	I find that I could not cope with all the work that I had to do during my internship.	0.5464	
	q8	I am angered because of things that were outside of my control.	0.5301	
	q9	I feel that difficulties at work are piling up so high that I could not overcome them.	0.6119	
	q10	I have little interest or pleasure in doing things that I used to enjoy.	0.5282	Adapted from Patient Health Questionnaire 9
	q12	I have sleeping problems, either have trouble falling or staying asleep, or sleeping too much.	0.5987	
	q13	I feel tired or having little energy during my internship.	0.6674	
	q14	I have eating problems, either have poor appetite, or have been overeating.	0.6061	
	q15	I have trouble concentrating on things either work-related, or outside of my work.	0.5766	
	q27	I am not as productive at work because I am losing sleep over work-related traumatic experiences.	0.5557	Adapted from Professional Quality of Life Scale
	q28	I feel trapped by my job as a medical intern.	0.6573	
	q29	I feel worn out because of my work as a medical intern.	0.7137	
	q30	I feel overwhelmed because my case workload seems endless during the internship.	0.7899	
	q31	I feel bogged down and held back by the internship hospital.	0.6036	
	q32	I am preoccupied by concerns about multiple patients during my internship.	0.6298	
	q33	I find it difficult to separate my personal life from my life as a medical intern.	0.6974	
	q34	I have been affected by the hardship and stress experienced by those patients I help.	0.6623	
	q35	I have felt on edge about various things because of my work helping patients.	0.6797	
	q36	I avoid certain activities or situations because they remind me of frightening experiences of the patients I help.	0.5561	
	q37	As a result of my helping, I have intrusive, frightening thoughts.	0.51	
	q79	I feel emotionally vulnerable within my internship hospital environment	0.4576	New
	q81	I get bullied or victimised within my internship hospital.	0.3678	New
	q19	I like my work as a medical intern.	0.5018	

Factor 3 – Job satisfaction (n=8) (Cronbach's alpha 0.90)	q20	My ability to keep up with clinical techniques and protocols makes me feel pleased.	0.6179	Adapted from Professional Quality of Life Scale
	q21	My internship work makes me feel satisfied.	0.6173	
	q22	I believe I can make a difference through my work.	0.7568	
	q23	I am proud of what I can do to help as a medical intern.	0.7939	
	q24	I believe that I am a success as a medical intern.	0.6805	
	q25	I am happy that I chose to do this work.	0.6637	
	q26	My internship experience met my expectation.	0.4044	New

Table a. Removing MIES items with cross-loading and high communality

Variable	Factor1	Factor2	Factor3	Uniqueness
q84	0.7175	0.0309	0.0424	0.4425
q88	0.6737	0.1285	-0.2806	0.5392
q63	0.6635	0.1232	-0.1118	0.5289
q45	0.6627	-0.0208	0.0993	0.5156
q59	0.6582	-0.0308	0.1249	0.5097
q82	0.6537	0.0205	-0.0053	0.5642
q86	0.6466	0.1267	-0.3657	0.5641
q44	0.6391	0.0163	0.1271	0.5077
q50	0.6348	-0.0293	0.2685	0.4217
q65	0.6337	0.3028	-0.3205	0.4559
q49	0.6313	-0.0893	0.2237	0.4989
q62	0.6225	-0.0051	0.1213	0.5467
q47	0.6171	-0.0304	0.2012	0.5076
q85	0.6165	0.0805	-0.2248	0.6336
q73	0.6076	0.0184	0.0837	0.5775
q83	0.6045	0.0322	-0.0052	0.6206
q66	0.602	0.3619	-0.3406	0.4392
q43	0.5948	-0.0428	0.2375	0.5132
q39	0.5943	-0.0111	0.2288	0.5041
q87	0.5912	0.1691	-0.3337	0.6064
q74	0.5728	-0.0417	0.1722	0.5933
q40	0.5724	0.1825	-0.1577	0.6136
q78	0.5711	0.033	0.1365	0.581
q77	0.5698	-0.011	0.1819	0.5742
q64	0.5535	0.0342	-0.171	0.7194
q41	0.547	0.066	0.2074	0.5359
q76	0.5426	-0.0462	0.1219	0.6645
q72	0.5383	0.0604	-0.0305	0.6929
q46	0.5161	-0.0726	0.2611	0.6048
q53	0.5082	0.098	0.2499	0.5252
q38	0.5024	-0.0915	0.3293	0.5668
q55	0.4975	-0.1386	0.1411	0.7292
q57	0.4674	-0.0706	0.2924	0.6318
q42	0.4634	-0.0431	0.2916	0.6251
q51	0.4128	-0.1361	0.3694	0.6401
q48	0.3961	-0.1416	0.2992	0.7184
q80	0.3379	0.0782	0.2146	0.7511
q75	0.2934	0.1707	0.1221	0.7924
q18	0.2198	0.2166	0.1703	0.7895
q30	0.1303	0.7755	-0.2018	0.3736
q29	0.1497	0.7058	-0.1249	0.4468
q33	0.035	0.6776	-0.1279	0.5596
q13	0.0675	0.6698	0.0368	0.4934

q28	0.048	0.6584	0.0505	0.5152
q2	0.141	0.653	-0.036	0.4977
q35	-0.0492	0.6528	-0.0177	0.6027
q11	-0.0253	0.6337	0.2913	0.4205
q34	-0.0048	0.6261	-0.1042	0.6381
q9	-0.0472	0.6216	0.193	0.532
q1	0.1191	0.6205	-0.1879	0.5929
q14	-0.044	0.6197	0.1249	0.5776
q12	-0.0201	0.6146	0.086	0.5939
q31	0.1892	0.5986	0.0409	0.4951
q32	0.0559	0.5983	-0.2118	0.6519
q15	-0.089	0.5946	0.2683	0.53
q27	-0.136	0.5614	0.2659	0.5924
q36	-0.2327	0.5545	0.2306	0.6487
q10	-0.0754	0.55	0.1725	0.6473
q5	0.0632	0.547	0.1116	0.6156
q8	0.0696	0.5257	0.0003	0.6899
q37	-0.1978	0.5143	0.2875	0.646
q4	-0.0644	0.5128	0.2537	0.6285
q79	0.0758	0.4709	0.0994	0.7012
q16	-0.1433	0.4038	0.3159	0.7182
q81	0.0921	0.3891	0.1884	0.7202
q70	0.0852	0.2842	0.1789	0.8197
q68	0.1871	0.2739	0.0802	0.8194
q3	0.0209	0.2721	0.2548	0.811
q23	-0.0881	0.003	0.7665	0.4518
q22	-0.0299	-0.0205	0.7335	0.4849
q24	-0.0063	0.0444	0.6502	0.5611
q25	0.0479	0.1733	0.6226	0.4877
q20	0.0766	0.0189	0.6156	0.5731
q21	0.1254	0.1556	0.5819	0.4999
q52	0.3175	-0.1561	0.5414	0.5485
q61	0.3471	-0.0926	0.4667	0.5887
q58	0.4381	-0.0847	0.4567	0.5017
q19	0.1031	0.2888	0.4565	0.5617
q60	0.4074	-0.1223	0.4084	0.6026
q26	0.2883	0.1783	0.3882	0.5726
q69	0.099	0.1882	0.2858	0.8061
q7	0.0464	0.2075	0,2649	0.8354

Variable	Factor1	Factor2	Factor3	Uniqueness
q84	0.7155	0.0285	0.0502	0.4433
q88	0.6774	0.1186	-0.2699	0.5388
q63	0.6639	0.1198	-0.1008	0.528

q45	0.6566	-0.0177	0.1103	0.5155
q59	0.653	-0.0288	0.1329	0.5103
q82	0.6529	0.0177	0.001	0.5642
q86	0.6502	0.1172	-0.353	0.5643
q65	0.6403	0.2909	-0.3076	0.4556
q44	0.6326	0.0211	0.1391	0.5067
q50	0.6262	-0.023	0.2783	0.4218
q49	0.623	-0.0825	0.2322	0.4985
q85	0.62	0.0723	-0.2181	0.6322
q62	0.6148	0.0007	0.1345	0.5456
q66	0.6109	0.3484	-0.3294	0.4381
q47	0.6106	-0.026	0.2093	0.5078
q73	0.6059	0.017	0.0889	0.5783
q83	0.6045	0.0289	0.0002	0.6205
q87	0.5973	0.158	-0.3245	0.6048
q39	0.5874	-0.006	0.237	0.5046
q43	0.585	-0.0351	0.2497	0.5119
q40	0.5776	0.1738	-0.1516	0.6128
q77	0.5668	-0.0091	0.1857	0.5751
q74	0.5656	-0.0362	0.1815	0.5928
q78	0.565	0.0377	0.1472	0.5803
q64	0.5516	0.031	-0.1594	0.7207
q41	0.5419	0.0691	0.2159	0.5367
q76	0.5382	-0.0438	0.1279	0.6645
q72	0.5373	0.0601	-0.0219	0.6918
q46	0.5081	-0.0639	0.2676	0.6042
q53	0.5051	0.1013	0.2552	0.5267
q38	0.4931	-0.0831	0.3353	0.5672
q55	0.4881	-0.1316	0.1512	0.7288
q57	0.4576	-0.0614	0.2999	0.6315
q42	0.4542	-0.0343	0.2998	0.6242
q58	0.4277	-0.0716	0.4608	0.5019
q51	0.4017	-0.1232	0.3749	0.639
q60	0.3961	-0.1088	0.4143	0.6008
q48	0.388	-0.1344	0.3018	0.7189
q61	0.3379	-0.0794	0.4678	0.5897
q80	0.3373	0.0799	0.2146	0.7536
q52	0.3046	-0.1382	0.5434	0.548
q75	0.2969	0.1637	0.1203	0.7984
q26	0.2825	0.1889	0.3949	0.5705
q18	0.2182	0.2223	0.1778	0.7867
q31	0.1948	0.5946	0.0523	0.4947

q29	0.1587	0.6995	-0.111	0.4427
q2	0.1511	0.6468	-0.0279	0.4967
q30	0.1433	0.765	-0.1895	0.3713
q1	0.1327	0.6085	-0.1821	0.5935
q21	0.1176	0.1719	0.5852	0.4975
q81	0.1013	0.3787	0.1821	0.7348
q19	0.1008	0.2989	0.459	0.5621
q79	0.0866	0.4605	0.0968	0.7115
q8	0.0745	0.5227	0.0112	0.6881
q13	0.0737	0.6686	0.0491	0.4881
q5	0.0722	0.5443	0.1146	0.617
q20	0.0668	0.0377	0.6164	0.5711
q32	0.0627	0.5906	-0.1958	0.6506
q28	0.0556	0.6553	0.0598	0.5134
q33	0.0456	0.669	-0.1177	0.5589
q25	0.04	0.191	0.6251	0.4849
q34	0.002	0.6202	-0.0904	0.636
q12	-0.0143	0.6142	0.095	0.5903
q24	-0.0174	0.0658	0.6518	0.5567
q11	-0.0225	0.6394	0.3008	0.4143
q14	-0.0364	0.6173	0.1307	0.5782
q9	-0.0392	0.621	0.1965	0.5335
q35	-0.0408	0.6472	-0.009	0.6022
q22	-0.0418	0.0005	0.732	0.4837
q4	-0.0597	0.5145	0.2571	0.6297
q10	-0.0689	0.5494	0.1765	0.6482
q15	-0.0857	0.599	0.276	0.5258
q23	-0.0986	0.0232	0.762	0.4542
q27	-0.1306	0.5632	0.2686	0.5938
q16	-0.1421	0.4078	0.319	0.7186
q37	-0.195	0.5174	0.2912	0.6459
q36	-0.2289	0.5564	0.2357	0.6472

Table b. MIES factors and item loading (Promax rotation)

Variable	Factor1	Factor2	Factor3	Uniqueness
q84	0.7385	0.0156	0.0164	0.4364
q45	0.6818	-0.0233	0.0656	0.5154
q59	0.6773	-0.0366	0.0847	0.518
q82	0.6771	0.0043	-0.0316	0.5521
q50	0.6677	-0.0372	0.2211	0.4335

q88	0.6605	0.138	-0.295	0.5344
q44	0.6603	0.0105	0.1001	0.5042
q49	0.6579	-0.0948	0.1822	0.51
q63	0.6572	0.1152	-0.1077	0.5339
q47	0.6427	-0.0361	0.1567	0.5183
q62	0.6419	-0.0144	0.0919	0.5493
q73	0.6359	-0.0055	0.0594	0.5705
q43	0.6251	-0.0516	0.1995	0.5189
q83	0.6249	0.0235	-0.0371	0.6109
q86	0.6232	0.1421	-0.3718	0.5639
q39	0.6171	-0.0233	0.2037	0.5101
q85	0.6083	0.0885	-0.2472	0.6281
q77	0.6047	-0.0351	0.1533	0.5703
q74	0.6034	-0.0509	0.1376	0.5903
q78	0.5966	0.0176	0.1159	0.575
q65	0.5787	0.2855	-0.2586	0.5228
q41	0.5709	0.0555	0.1799	0.5385
q87	0.5691	0.1779	-0.335	0.6098
q76	0.5662	-0.0573	0.0956	0.6624
q40	0.557	0.169	-0.1374	0.6281
q72	0.5524	0.0418	-0.0291	0.6842
q46	0.5441	-0.0704	0.2102	0.6216
q64	0.536	0.0298	-0.1612	0.732
q53	0.5353	0.0837	0.2125	0.538
q55	0.5152	-0.1479	0.1264	0.73
q42	0.4976	-0.0556	0.254	0.6343
q57	0.4929	-0.0705	0.2395	0.6575
q80	0.3672	0.0619	0.1857	0.7559
q30	0.0885	0.7899	-0.1747	0.3768
q29	0.1147	0.7137	-0.0933	0.4494
q33	0.0065	0.6974	-0.1178	0.5486
q35	-0.067	0.6797	-0.0263	0.5812
q13	0.0557	0.6674	0.0501	0.4948
q34	-0.0306	0.6623	-0.1165	0.6105
q2	0.1201	0.6581	-0.0214	0.4964
q28	0.0351	0.6573	0.0677	0.513
q1	0.084	0.63	-0.161	0.5991
q32	0.0145	0.6298	-0.1975	0.6374
q9	-0.0368	0.6119	0.1914	0.5366
q14	-0.0322	0.6061	0.1229	0.5881
q31	0.1777	0.6036	0.0415	0.4917
q12	-0.0141	0.5987	0.0953	0.604

q15	-0.0649	0.5766	0.2625	0.5408
q36	-0.2186	0.5561	0.2183	0.6513
q27	-0.121	0.5557	0.2581	0.5956
q5	0.0608	0.5464	0.1121	0.6139
q8	0.057	0.5301	0.0148	0.6847
q10	-0.0539	0.5282	0.165	0.6652
q37	-0.172	0.51	0.2673	0.6559
q4	-0.0483	0.4961	0.2561	0.6334
q79	0.0802	0.4576	0.0978	0.7102
q81	0.1112	0.3678	0.1662	0.7395
q23	-0.0204	-0.0641	0.7939	0.407
q22	0.0332	-0.0815	0.7568	0.4445
q24	0.0504	-0.0106	0.6805	0.5169
q25	0.1001	0.1108	0.6637	0.4377
q20	0.1379	-0.0301	0.6179	0.5578
q21	0.1712	0.0978	0.6173	0.4586
q19	0.1379	0.2313	0.5018	0.5299
q26	0.3177	0.1422	0.4044	0.5569

Table c. Confirmatory factor analysis for 3-factor structure

Country	RMSEA (<=0.06 or 0.08)	CFI (>=0.95 or 0.90)	SRMR (<=0.08 or 0.10)	Cronbach's alpha (>0.80)	Cronbach's alpha for factor 1	Cronbach's alpha for factor 2	Cronbach's alpha for factor 3
**Overall (all sample)**	**0.063**	**0.772**	**0.067**	**0.96**	**0.95**	**0.94**	**0.90**
**Multigroup (4 country only)**	**0.081**	**0.643**	**0.103**	**0.96**	**0.95**	**0.94**	**0.91**
Kenya	0.071	0.765	0.073	0.97	0.96	0.94	0.91
Uganda	0.067	0.725	0.078	0.95	0.94	0.93	0.87
Vietnam	0.087	0.641	0.087	0.96	0.95	0.93	0.91
China	0.086	0.674	0.088	0.96	0.95	0.94	0.93

Supplementary appendix 7. Six-factor structure for MIES analysis

Table a. Remove cross loading

Variable	Factor1	Factor2	Factor3	Factor4	Factor5	Factor6	Uniqueness
q43	0.9584	-0.1154	-0.1802	0.0438	0.0033	-0.0136	0.4136
q50	0.9240	-0.0837	-0.1294	0.0291	0.0760	-0.0522	0.3406
q47	0.9130	-0.0543	-0.2010	0.0451	0.0171	-0.0164	0.4215
q45	0.8894	0.0139	-0.2208	0.0909	-0.0483	-0.0886	0.4346
q41	0.7839	-0.0950	-0.1518	0.0870	0.1852	-0.0879	0.4600
q49	0.7589	0.0526	-0.0292	-0.0123	-0.0500	-0.0667	0.4595
q46	0.7084	0.0123	-0.0182	-0.0017	-0.1067	0.0475	0.5598
q51	0.6945	-0.0801	0.0529	-0.0801	-0.1110	0.1203	0.5889
q48	0.6916	-0.1252	-0.0389	-0.1028	0.0171	0.0438	0.6711
q39	0.6744	0.0464	0.0069	0.0344	0.0304	-0.0936	0.4751
q38	0.6727	-0.0691	0.0847	-0.0589	0.0640	-0.0795	0.5368
q42	0.6324	-0.0215	0.0308	-0.0063	-0.0154	0.0217	0.5976
q53	0.5862	-0.0257	0.0218	0.0877	0.1977	-0.1218	0.4863
q52	0.5777	-0.1338	0.3198	-0.1166	-0.1456	0.1295	0.5163
q44	0.5750	0.2568	-0.0972	0.0538	-0.0737	0.0378	0.4866
q58	0.4690	0.0130	0.2584	-0.1195	0.0814	0.0215	0.4981
q62	0.3792	0.3329	0.0788	0.0258	-0.0799	-0.0735	0.5401
q61	0.3731	0.0422	0.2333	-0.1543	0.0524	0.1537	0.5723
q57	0.3434	0.1975	0.1397	-0.1035	0.0173	0.0580	0.6267
q55	0.3323	0.2253	0.1444	-0.0866	-0.1110	-0.1092	0.7157
q60	0.3138	0.1793	0.2863	-0.1408	-0.1115	0.1419	0.5830
q88	-0.0559	0.8416	-0.1698	0.1221	-0.1728	0.0207	0.4908
q82	-0.1086	0.8027	-0.0061	-0.1079	0.0481	0.1148	0.4671
q86	-0.0223	0.7907	-0.1841	0.1851	-0.2717	-0.0594	0.5157
q72	-0.1902	0.7307	0.0732	-0.0356	-0.0190	0.0636	0.6192
q87	-0.0412	0.7087	-0.1592	0.2033	-0.1740	-0.0938	0.5769
q83	-0.0268	0.6935	-0.0437	-0.0722	0.0365	0.1119	0.5533
q85	0.0465	0.6558	-0.1329	0.0946	-0.1210	-0.0631	0.6160
q84	0.1173	0.6334	-0.0012	-0.0617	0.1151	-0.0186	0.4110
q73	-0.0108	0.6215	-0.0072	-0.1404	0.2191	0.0681	0.4916
q76	-0.0506	0.5946	0.0989	-0.1722	0.0983	0.0796	0.5980
q78	0.0050	0.5853	0.1233	-0.0703	0.0433	0.0813	0.5342
q74	0.0480	0.5785	0.0598	-0.1700	0.0843	0.1578	0.5170
q65	0.0137	0.5626	-0.0520	0.3379	0.0203	-0.3722	0.3753
q77	-0.0114	0.5566	0.1200	-0.1661	0.1994	0.0585	0.5019
q66	-0.0026	0.5338	-0.0743	0.3891	0.0590	-0.3830	0.3536
q64	0.0946	0.4860	-0.0094	0.0860	-0.1153	-0.1995	0.6909
q63	0.2287	0.4722	0.0016	0.1722	-0.0712	-0.2221	0.5007
q59	0.2649	0.4558	0.0511	-0.0719	0.0381	-0.0263	0.5036
q80	-0.0884	0.3778	0.1694	-0.0718	0.1774	0.1062	0.6952
q40	0.2229	0.3411	-0.0557	0.2207	0.0547	-0.3007	0.5705

q23	-0.1743	-0.1807	0.9677	-0.1036	0.0580	-0.0050	0.3769
q22	-0.1211	-0.1218	0.9209	-0.1002	-0.0296	0.0293	0.4181
q25	-0.1262	-0.0387	0.8824	0.1192	-0.0679	-0.0327	0.3882
q21	-0.0993	0.0201	0.8548	0.1123	-0.0760	-0.0706	0.3916
q24	-0.0194	-0.1272	0.8341	0.0274	-0.1636	0.0426	0.4859
q19	-0.2332	0.0809	0.7821	0.2158	0.0217	-0.1268	0.4375
q20	-0.0078	-0.0199	0.7033	-0.0413	-0.0847	0.1094	0.5378
q26	0.1558	0.0416	0.5345	0.1875	-0.0703	-0.1160	0.5108
q18	-0.1668	0.2202	0.4853	0.2034	-0.0506	-0.1836	0.6903
q7	0.0006	-0.0642	0.4066	0.2020	-0.0175	-0.0825	0.7975
q3	-0.0708	0.0031	0.3579	0.2328	-0.0114	0.0245	0.7950
q30	-0.1215	0.2252	-0.0634	0.7614	0.0020	-0.0257	0.3584
q29	-0.0960	0.1811	0.0646	0.7058	-0.0068	-0.0988	0.4071
q13	0.0883	-0.0347	0.0886	0.6810	-0.0177	-0.0074	0.4658
q33	0.0119	0.1360	-0.1795	0.6773	-0.1183	0.2189	0.5369
q2	0.1084	0.0001	0.0461	0.6741	0.0249	-0.1081	0.4533
q1	-0.0061	0.0811	-0.0750	0.6315	0.0633	-0.1388	0.5587
q32	0.0592	0.1429	-0.2897	0.6199	-0.1295	0.1971	0.6237
q28	-0.0499	0.0527	0.1140	0.6188	0.0353	0.0415	0.5076
q34	0.0175	0.2055	-0.2842	0.6125	-0.2309	0.4663	0.5165
q11	0.1657	-0.2067	0.2177	0.5992	0.0465	0.1164	0.4083
q35	-0.0201	0.1377	-0.2088	0.5947	-0.1085	0.4466	0.5132
q31	0.1918	0.0259	-0.0256	0.5809	0.0665	0.0255	0.4850
q9	0.0277	-0.0909	0.1483	0.5677	0.0420	0.1521	0.5302
q12	0.0561	-0.0678	0.0099	0.5676	0.0723	0.1283	0.5900
q5	0.0794	-0.0328	0.1260	0.5336	0.0089	0.0329	0.6048
q14	0.0086	-0.0433	-0.0171	0.5221	0.1452	0.2134	0.5633
q15	0.0035	-0.0797	0.1680	0.5163	0.0147	0.2775	0.5197
q8	0.0317	0.0592	0.0035	0.5143	-0.0227	0.0747	0.6858
q10	0.1020	-0.1523	0.0336	0.4963	0.0737	0.1879	0.6377
q27	0.0021	-0.1103	0.0853	0.4547	0.0820	0.3338	0.5641
q4	-0.0121	-0.1177	0.2513	0.4503	0.0716	0.0992	0.6263
q70	0.0477	-0.1160	-0.1305	0.0299	0.7658	-0.0348	0.5492
q69	0.0490	-0.1172	-0.0119	-0.0719	0.7419	-0.0215	0.5551
q81	0.0627	-0.0786	-0.0931	0.1677	0.6187	0.0359	0.5489
q68	0.0803	0.0125	-0.1008	0.1160	0.4991	-0.0706	0.7115
q75	0.0247	0.2106	-0.0594	-0.0079	0.4177	0.0330	0.6969
q79	0.0028	-0.0101	-0.0514	0.3167	0.4084	0.0309	0.6367
q36	-0.1130	-0.0009	-0.0437	0.4147	-0.0074	0.5919	0.4967
q37	-0.0725	-0.0078	-0.0037	0.3700	0.0032	0.5901	0.4936
q16	-0.0977	-0.0429	0.1459	0.2623	0.1044	0.3654	0.6676

Variable	Factor1	Factor2	Factor3	Factor4	Factor5	Factor6	Uniqueness
q43	0.9236	-0.0830	0.0104	-0.1219	-0.0289	-0.0522	0.4010
q50	0.8834	-0.0529	0.0006	-0.0626	0.0322	-0.0163	0.3350
q47	0.8812	-0.0418	0.0075	-0.1347	-0.0037	-0.0199	0.4082

q45	0.8630	-0.0818	0.0561	-0.1381	-0.0394	0.1094	0.4197
q41	0.7499	-0.0621	0.0736	-0.0828	0.1067	-0.0136	0.4556
q49	0.7403	0.0429	-0.0446	0.0016	-0.0667	0.0427	0.4527
q46	0.6732	0.0104	-0.0356	0.0205	-0.0570	0.0048	0.5668
q48	0.6667	-0.0572	-0.1433	0.0046	0.0260	-0.0998	0.6715
q39	0.6554	0.0031	-0.0027	0.0700	0.0238	0.0842	0.4674
q38	0.6421	-0.0214	-0.0957	0.1240	0.0331	-0.0086	0.5381
q51	0.6125	-0.0119	-0.1169	0.0928	-0.0172	-0.0627	0.6353
q42	0.6052	0.0343	-0.0317	0.0530	-0.0306	-0.0571	0.5982
q53	0.5635	0.0136	0.0933	0.0398	0.1081	0.0177	0.4984
q44	0.5502	0.2576	0.0417	-0.0792	-0.0886	0.0465	0.4837
q58	0.4264	0.1137	-0.1240	0.2549	0.0814	-0.0472	0.5312
q82	-0.0999	0.8178	-0.0792	-0.0262	0.0140	0.1672	0.4599
q73	0.0003	0.7629	-0.0971	-0.0502	0.0694	0.0365	0.4710
q74	0.0269	0.7555	-0.1336	0.0038	-0.0065	-0.0167	0.4940
q76	-0.0648	0.7310	-0.1479	0.0460	0.0142	0.0448	0.5767
q77	-0.0074	0.7183	-0.1326	0.0658	0.0686	0.0206	0.4825
q72	-0.1484	0.7060	0.0140	0.0124	-0.0701	0.1734	0.6094
q78	0.0081	0.6773	-0.0241	0.0550	-0.0479	0.0538	0.5223
q83	-0.0180	0.6376	-0.0536	-0.0234	0.0589	0.1964	0.5591
q84	0.1165	0.6288	-0.0358	-0.0081	0.0477	0.1826	0.4099
q88	-0.0208	0.4828	0.1487	-0.1065	-0.0200	0.4760	0.4679
q59	0.2520	0.4378	-0.0606	0.0494	0.0160	0.1414	0.5163
q75	-0.0083	0.3650	0.0172	-0.0289	0.2955	-0.0289	0.6956
q30	-0.1221	-0.0640	0.8393	-0.0380	0.0721	0.2800	0.3408
q13	0.0547	-0.0306	0.7898	-0.0136	-0.1102	-0.0126	0.4374
q29	-0.1083	-0.0702	0.7874	0.0626	0.0368	0.2690	0.3878
q2	0.0877	-0.1231	0.7505	0.0068	-0.0223	0.1385	0.4626
q1	-0.0155	-0.0656	0.7165	-0.1125	0.0037	0.1670	0.5699
q33	-0.0211	-0.0360	0.7104	-0.1394	0.0290	0.0794	0.5784
q11	0.1396	-0.0488	0.6976	0.0899	-0.0709	-0.2110	0.3705
q12	0.0386	0.1030	0.6770	-0.1175	-0.1065	-0.2036	0.5295
q28	-0.0492	-0.1073	0.6731	0.1262	0.0912	0.1278	0.5004
q32	0.0072	-0.0534	0.6409	-0.2086	0.0281	0.1160	0.6654
q9	0.0002	-0.0445	0.6368	0.0890	0.0341	-0.0903	0.5311
q14	-0.0241	0.2063	0.6349	-0.1450	-0.0427	-0.2755	0.4913
q31	0.1610	-0.1191	0.6323	0.0135	0.1195	0.1233	0.4663
q15	-0.0360	0.1368	0.6054	0.0445	-0.0618	-0.2692	0.4827
q5	0.0613	-0.0400	0.6001	0.0592	-0.0226	-0.0031	0.6033
q10	0.0743	0.0365	0.5739	-0.0661	-0.0398	-0.2415	0.6051
q8	0.0030	-0.0022	0.5623	-0.0102	0.0006	0.0473	0.6875
q37	-0.0918	0.0720	0.3603	0.0662	0.1599	-0.2201	0.7026
q23	-0.1498	-0.0391	-0.1391	0.8964	0.1120	-0.1250	0.3587
q22	-0.0917	0.0057	-0.1401	0.8391	0.0378	-0.1165	0.4111
q25	-0.0806	-0.0311	0.1047	0.8064	-0.0047	0.0079	0.3727
q21	-0.0413	0.0096	0.1079	0.7660	-0.0398	0.0328	0.3809

q24	0.0050	-0.0791	-0.0105	0.7634	-0.0539	-0.0743	0.4790
q19	-0.1652	0.0324	0.2316	0.6959	0.0236	0.0952	0.4367
q20	0.0061	0.1319	-0.0556	0.6147	-0.0520	-0.1373	0.5362
q26	0.1785	-0.0785	0.1776	0.5187	0.0025	0.1446	0.4979
q18	-0.1099	0.1849	0.2486	0.3621	-0.1485	0.1108	0.7090
q7	0.0360	-0.0864	0.2044	0.3418	-0.0227	0.0336	0.8096
q69	-0.0118	0.0523	-0.0723	0.1150	0.6667	-0.0819	0.5027
q70	-0.0026	0.0269	0.0390	0.0015	0.6623	-0.0713	0.5100
q68	0.0498	0.0110	0.1096	0.0250	0.4571	0.0618	0.6876
q81	0.0402	0.0692	0.2078	-0.0090	0.4488	-0.1031	0.5867
q86	0.0159	0.3366	0.1967	-0.1041	-0.0834	0.5526	0.4802
q87	0.0056	0.3281	0.2278	-0.1137	-0.0611	0.4753	0.5604
q85	0.0803	0.2939	0.1088	-0.0562	0.0234	0.4582	0.5769
q63	0.2388	0.2755	0.1876	-0.0080	-0.0933	0.3157	0.5382
q64	0.1253	0.2569	0.0838	-0.0116	-0.0950	0.3143	0.7288

Variable	Factor1	Factor2	Factor3	Factor4	Factor5	Factor6	Uniqueness
q43	0.9133	-0.0922	-0.0743	-0.0943	0.0553	-0.0148	0.3983
q45	0.8902	-0.0430	0.1119	-0.1520	-0.0908	-0.0734	0.4198
q47	0.8799	-0.0388	-0.0402	-0.1208	0.0237	-0.0037	0.4028
q50	0.8688	-0.0422	-0.0222	-0.0444	-0.0092	0.0421	0.3351
q49	0.7414	0.0689	-0.0005	-0.0012	-0.0605	-0.0838	0.4549
q41	0.7369	-0.0471	0.0877	-0.0613	-0.0447	0.1008	0.4542
q46	0.6805	0.0093	-0.1149	0.0240	0.0676	-0.0537	0.5623
q39	0.6744	0.0417	0.1027	0.0579	-0.1389	-0.0039	0.4615
q48	0.6280	-0.0661	-0.1797	0.0419	0.0104	0.0456	0.6810
q38	0.6212	0.0111	-0.0393	0.1378	-0.0867	0.0217	0.5389
q42	0.5931	0.0190	-0.1488	0.0641	0.1101	0.0001	0.5962
q51	0.5898	-0.0139	-0.1768	0.1135	0.0385	-0.0001	0.6371
q44	0.5673	0.2450	-0.0318	-0.0864	0.1051	-0.0842	0.4840
q53	0.5507	0.0364	0.1272	0.0427	-0.0500	0.1038	0.5021
q82	-0.0605	0.8043	0.0345	-0.0773	0.0037	-0.0047	0.4537
q73	0.0029	0.7225	-0.0373	-0.0532	0.0594	0.0557	0.4689
q74	0.0010	0.7104	-0.0811	0.0289	0.0582	0.0016	0.4907
q76	-0.0680	0.6984	-0.0226	0.0474	-0.0184	-0.0039	0.5713
q77	-0.0140	0.6835	-0.0574	0.0693	0.0337	0.0534	0.4815
q72	-0.0894	0.6814	0.1083	-0.0298	0.0118	-0.0993	0.6150
q78	0.0055	0.6485	0.0015	0.0531	0.0762	-0.0443	0.5196
q83	0.0424	0.6303	0.0593	-0.0787	-0.0265	0.0269	0.5701
q84	0.1552	0.6285	0.0442	-0.0607	0.0081	0.0321	0.4207
q59	0.2673	0.4597	0.0614	0.0089	-0.0698	-0.0052	0.5213
q30	-0.0738	0.0405	1.0026	-0.0800	-0.1604	-0.0259	0.3021
q29	-0.0640	0.0365	0.9378	0.0182	-0.1493	-0.0528	0.3539
q28	-0.0392	-0.0285	0.7333	0.1142	-0.0657	0.0295	0.4788
q32	0.0231	-0.0104	0.7001	-0.1987	-0.0510	-0.0289	0.6397
q31	0.1690	-0.0495	0.6974	0.0136	-0.0756	0.0586	0.4439

q33	-0.0081	-0.0064	0.6616	-0.1273	0.0658	-0.0161	0.5732
q1	0.0164	-0.0032	0.6556	-0.1514	0.0783	-0.0348	0.5731
q2	0.1094	-0.0580	0.6485	-0.0244	0.1068	-0.0509	0.4676
q5	0.0438	-0.0063	0.4719	0.0712	0.1491	-0.0411	0.6014
q8	0.0119	0.0105	0.4233	-0.0076	0.1634	-0.0114	0.6880
q9	-0.0392	-0.0383	0.3875	0.1276	0.2814	0.0360	0.5353
q23	-0.1692	-0.0545	-0.1513	0.9408	-0.0324	0.1199	0.3504
q22	-0.1017	-0.0181	-0.1703	0.8774	-0.0019	0.0450	0.4068
q25	-0.0485	-0.0206	0.1170	0.8105	-0.0517	-0.0279	0.3758
q24	0.0052	-0.0837	-0.0429	0.8031	-0.0099	-0.0670	0.4667
q21	-0.0120	0.0261	0.1186	0.7611	-0.0422	-0.0542	0.3878
q19	-0.1345	0.0780	0.3223	0.6737	-0.1148	-0.0092	0.4449
q20	-0.0068	0.0922	-0.1733	0.6559	0.1165	-0.0288	0.5361
q26	0.2303	-0.0268	0.2557	0.4833	-0.1222	-0.0423	0.5158
q14	-0.0714	0.0920	-0.0611	-0.1000	0.8023	0.0772	0.4404
q12	0.0023	0.0189	0.0186	-0.0920	0.7425	0.0058	0.4763
q15	-0.0974	0.0514	0.0563	0.1129	0.6329	0.0163	0.4803
q10	0.0168	-0.0358	0.0490	-0.0042	0.5901	0.0369	0.5919
q11	0.0844	-0.0927	0.1796	0.1420	0.5676	-0.0059	0.3583
q13	0.0533	-0.0361	0.3924	-0.0222	0.4437	-0.0697	0.4311
q69	-0.0380	0.0304	-0.1330	0.1074	0.0462	0.7216	0.4747
q70	-0.0228	0.0018	-0.0583	-0.0086	0.0869	0.7213	0.4774
q81	0.0082	0.0491	0.1210	0.0101	0.0999	0.4726	0.5997
q68	0.0661	0.0277	0.2186	0.0096	-0.1282	0.4374	0.6931

Table b. MIES factors and item loading (Promax rotation)

Variable	Factor1	Factor2	Factor3	Factor4	Factor5	Factor6	Uniqueness
q43	0.9282	-0.0951	-0.0951	-0.1082	0.0726	-0.0178	0.3961
q45	0.9002	-0.0449	0.0941	-0.1583	-0.0789	-0.0732	0.4189
q47	0.8884	-0.0400	-0.0559	-0.1278	0.0390	-0.0095	0.4029
q50	0.8756	-0.0434	-0.0307	-0.0515	-0.0060	0.0443	0.3349
q49	0.7480	0.0684	-0.0062	-0.0108	-0.0582	-0.0799	0.4550
q41	0.7425	-0.0476	0.0768	-0.0657	-0.0381	0.1006	0.4541
q46	0.6792	0.0112	-0.1127	0.0258	0.0642	-0.0576	0.5630
q39	0.6715	0.0430	0.1076	0.0571	-0.1412	-0.0010	0.4612
q48	0.6299	-0.0647	-0.1795	0.0351	0.0162	0.0406	0.6818
q38	0.6117	0.0145	-0.0150	0.1457	-0.1190	0.0301	0.5364
q42	0.5944	0.0188	-0.1501	0.0595	0.1171	-0.0042	0.5958
q51	0.5832	-0.0113	-0.1626	0.1165	0.0296	-0.0029	0.6376
q44	0.5775	0.2432	-0.0497	-0.0955	0.1197	-0.0875	0.4824
q53	0.5529	0.0365	0.1221	0.0379	-0.0479	0.1084	0.5022
q82	-0.0663	0.8079	0.0392	-0.0713	-0.0085	-0.0050	0.4537
q73	0.0013	0.7259	-0.0406	-0.0541	0.0596	0.0520	0.4687
q74	-0.0114	0.7157	-0.0656	0.0401	0.0395	-0.0008	0.4898
q76	-0.0729	0.7028	-0.0134	0.0477	-0.0344	-0.0009	0.5714
q77	-0.0150	0.6862	-0.0560	0.0618	0.0342	0.0535	0.4827

q72	-0.0891	0.6845	0.0965	-0.0334	0.0238	-0.1019	0.6156
q78	-0.0037	0.6510	0.0108	0.0599	0.0679	-0.0447	0.5194
q83	0.0354	0.6336	0.0621	-0.0714	-0.0304	0.0229	0.5705
q84	0.1512	0.6307	0.0487	-0.0554	-0.0093	0.0370	0.4205
q59	0.2555	0.4637	0.0784	0.0220	-0.0958	-0.0003	0.5197
q30	-0.0841	0.0414	0.9695	-0.0589	-0.1202	-0.0294	0.3039
q29	-0.0781	0.0371	0.9127	0.0393	-0.1158	-0.0506	0.3553
q28	-0.0525	-0.0274	0.7145	0.1285	-0.0259	0.0233	0.4746
q31	0.1591	-0.0482	0.6762	0.0289	-0.0424	0.0523	0.4421
q32	0.0128	-0.0098	0.6727	-0.1735	-0.0166	-0.0406	0.6352
q33	-0.0159	-0.0069	0.6303	-0.1070	0.1104	-0.0318	0.5648
q1	0.0148	-0.0072	0.6272	-0.1341	0.1011	-0.0351	0.5733
q2	0.1045	-0.0613	0.6261	-0.0068	0.1238	-0.0466	0.4709
q5	0.0414	-0.0034	0.4529	0.0842	0.1557	-0.0425	0.6140
q8	0.0148	0.0114	0.4013	0.0044	0.1638	-0.0106	0.7027
q23	-0.1971	-0.0420	-0.0915	0.9347	-0.0464	0.1153	0.3500
q22	-0.1359	-0.0059	-0.1091	0.8838	-0.0213	0.0409	0.3943
q24	-0.0294	-0.0727	0.0101	0.8144	-0.0184	-0.0741	0.4508
q25	-0.0642	-0.0125	0.1440	0.7934	-0.0366	-0.0269	0.3883
q21	-0.0155	0.0314	0.1302	0.7269	-0.0126	-0.0499	0.4133
q20	-0.0180	0.0985	-0.1474	0.6422	0.1211	-0.0316	0.5383
q26	0.2166	-0.0220	0.2712	0.4801	-0.1144	-0.0379	0.5192
q14	-0.0540	0.0782	-0.0931	-0.0980	0.8151	0.0754	0.4456
q12	0.0172	0.0046	-0.0133	-0.0891	0.7592	0.0070	0.4786
q15	-0.0881	0.0445	0.0297	0.1116	0.6599	0.0079	0.4790
q10	0.0283	-0.0433	0.0189	-0.0043	0.6175	0.0274	0.5895
q11	0.0911	-0.0983	0.1558	0.1439	0.5873	-0.0094	0.3659
q69	-0.0443	0.0311	-0.1182	0.1063	0.0307	0.7257	0.4738
q70	-0.0201	-0.0004	-0.0612	-0.0152	0.0845	0.7253	0.4759
q81	0.0050	0.0483	0.1148	0.0127	0.1101	0.4693	0.5999
q68	0.0578	0.0292	0.2233	0.0157	-0.1311	0.4395	0.6925

Supplementary appendix 8. Comparison of MIES aggregate scores between different level facilities in Kenya

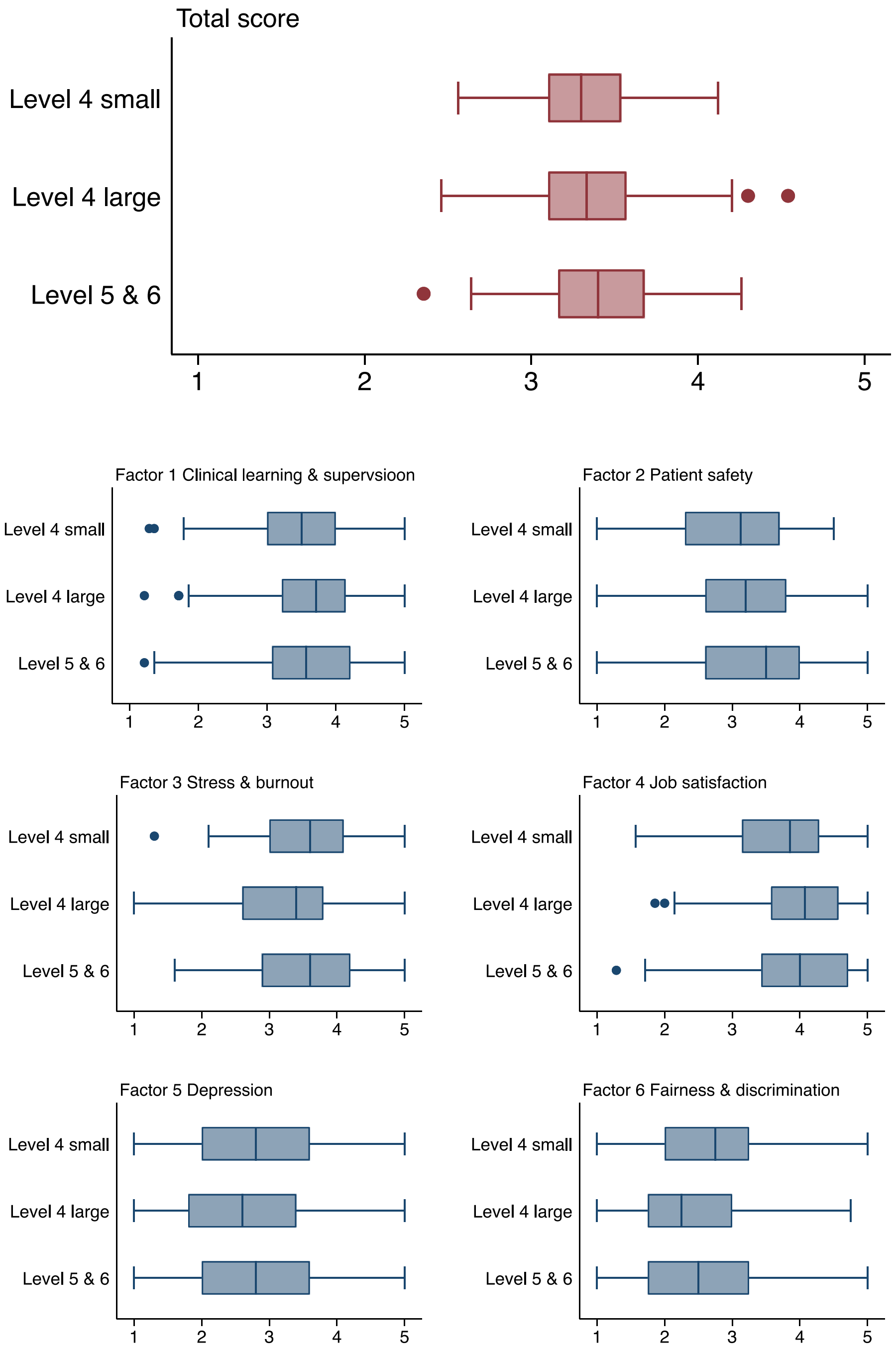

Note: Comparison of individuals interned in different facilities. 99 participants interned in level 4 small facilities (bed number < 196), 98 interned in level 4 large facilities (bed number≥196), 137 interned in level 5 & 6 facilities, and 24 interned in other types of facilities which is not shown here. All "negative" items have been reversed and higher score in each factor is more favourable.

Supplementary appendix 9. Comparison of response to each item by year of completing internship

Note: Proportion that selected "agree" and "strongly agree" or "often" and "very often" from the questionnaire survey.

Item	Overall (n=1646)	Current intern (n=650)	Completed internship after 2020 (n=820)	Completed internship between 2018-2019 (n=176)
Q1	46%	38%	52%	50%
Q2	44%	38%	48%	48%
Q3	51%	53%	49%	51%
Q4	24%	22%	25%	23%
Q5	25%	24%	25%	30%
Q6	56%	57%	56%	53%
Q7	47%	48%	46%	46%
Q8	34%	33%	35%	38%
Q9	18%	17%	19%	21%
Q10	23%	26%	22%	21%
Q11	20%	23%	17%	18%
Q12	28%	27%	28%	26%
Q13	35%	33%	35%	35%
Q14	25%	24%	26%	27%
Q15	16%	19%	14%	18%
Q16	6%	8%	5%	5%
Q17	82%	79%	84%	85%
Q18	34%	35%	33%	34%
Q19	58%	61%	55%	55%
Q20	73%	68%	76%	77%
Q21	58%	56%	60%	60%
Q22	72%	67%	75%	77%
Q23	75%	71%	78%	78%
Q24	68%	59%	73%	74%
Q25	64%	62%	65%	65%
Q26	47%	41%	51%	52%
Q27	15%	18%	14%	13%
Q28	26%	28%	25%	25%
Q29	40%	35%	42%	46%
Q30	39%	31%	42%	49%
Q31	30%	29%	30%	30%
Q32	41%	38%	43%	47%
Q33	32%	27%	34%	40%
Q34	25%	24%	25%	28%
Q35	22%	18%	26%	25%
Q36	13%	12%	13%	12%
Q37	9%	9%	8%	13%
Q38	65%	60%	67%	76%
Q39	56%	54%	56%	58%
Q40	29%	33%	27%	26%
Q41	58%	57%	58%	61%
Q42	64%	55%	69%	71%
Q43	57%	53%	61%	56%
Q44	54%	51%	57%	55%
Q45	46%	41%	48%	55%

Q46	61%	55%	64%	69%
Q47	61%	60%	61%	62%
Q48	72%	68%	74%	74%
Q49	54%	53%	53%	59%
Q50	56%	55%	57%	60%
Q51	72%	68%	75%	71%
Q52	70%	63%	75%	76%
Q53	63%	66%	62%	60%
Q54	27%	33%	21%	30%
Q55	43%	37%	45%	50%
Q56	52%	50%	51%	60%
Q57	65%	59%	69%	64%
Q58	72%	65%	76%	76%
Q59	54%	54%	56%	52%
Q60	71%	67%	74%	76%
Q61	78%	74%	81%	80%
Q62	45%	40%	49%	46%
Q63	28%	29%	28%	30%
Q64	38%	35%	40%	34%
Q65	32%	34%	30%	32%
Q66	33%	38%	29%	31%
Q67	41%	34%	45%	44%
Q68	34%	32%	36%	34%
Q69	16%	16%	16%	16%
Q70	19%	17%	19%	20%
Q71	25%	26%	24%	25%
Q72	42%	39%	44%	48%
Q73	55%	54%	55%	59%
Q74	59%	57%	61%	61%
Q75	27%	25%	28%	28%
Q76	56%	57%	57%	52%
Q77	59%	58%	60%	60%
Q78	53%	50%	56%	52%
Q79	30%	30%	32%	26%
Q80	65%	65%	65%	67%
Q81	18%	16%	19%	17%
Q82	45%	44%	46%	44%
Q83	57%	56%	57%	60%
Q84	46%	45%	47%	45%
Q85	37%	34%	39%	38%
Q86	28%	29%	28%	24%
Q87	33%	37%	32%	27%
Q88	38%	37%	38%	38%

Supplementary appendix 10. Structured reflexivity statement

Domain	Question	Answer
Study conceptualisation	1. How does this study address local research and policy priorities?	This research is a collaboration between HIC and LMIC researchers. The research question is an issue of major priority in LMICs that have been reported in the literature.
	2. How were local researchers involved in study design?	The first author is a UK-based Chinese researcher (YZ) and the senior authors is a UK pediatrician with extensive experiences working in Kenya (ME). Together with a group of LMIC-based researchers, the research team co-designed the study including refining the study instrument and data interpretation to promote rigour.
Research management	3. How has funding been used to support the local research team(s)?	This project leveraged a wide range of funding from UK and elsewhere to support the research team including funding for the Kenyan, Uganda and Sierra Leone core researchers to develop skills and networks with an aim to develop further work in this area.
Data acquisition and analysis	4. How are research staff who conducted data collection acknowledged?	Key research staff involved in coordinating and collecting data have been listed as authors.
	5. How have members of the research partnership been provided with access to study data?	The first and senior authors have access to all data, each LMIC author have access to their own data as well as country-level aggregate data due to consideration for anonymity.
	6. How were data used to develop analytical skills within the partnership?	The first author who is an early career researcher have been supported by the senior author and the research group to develop analytical skill.
Data interpretation	7. How have research partners collaborated in interpreting study data?	All authors were involved in interpreting the study data, this included several rounds of discussion and feedback meetings held online.
Drafting and revising for intellectual content	8. How were research partners supported to develop writing skills?	The first author who is an early career researcher have been supported by the senior author and the research group to develop writing skill.
	9. How will research products be shared to address local needs?	While some of the findings have been preliminarily shared with key stakeholders in countries, we are planning to send this final publication again with policy stakeholders as a follow-up and also with research leaders in global health to advocate for more research in this area.
Authorship	10. How is the leadership, contribution and ownership of this work by LMIC researchers	We have included all LMIC researchers who are involved in study design, interpretation and reviewing the manuscript as authors. However, as this project hope to provide a globally

	recognised within the authorship?	relevant tool for all LMICs, we do acknowledge that both the first author and senior author are based in UK.
	11. How have early career researchers across the partnership been included within the authorship team?	The first author who is an early career researcher have been supported by the senior author and the research group to develop research skill.
	12. How has gender balance been addressed within the authorship?	Four authors are female (NM, NTBP, BW, CN), and 13 authors are male (YZ, SL, PKL, YKK, DM, MN, SQ, RS, KT, RT, FZ, DG, ME)
Training	13. How has the project contributed to training of LMIC researchers?	All early career researchers from LMIC been supported by first and senior authors and the research group to develop research skills. This included efforts to support each country to conduct analysis and draft their individual country reports. So far Vietnam has already produced their own report.
Infrastructure	14. How has the project contributed to improvements in local infrastructure?	This project has not directly contributed to improvements in local infrastructure, but we hope the findings will inform internship planning which will involve improvement in internship hospital infrastructure.
Governance	15. What safeguarding procedures were used to protect local study participants and researchers?	We were not aware of any significant risks to the participants of the study. There is a small risk that the potential sensitivity of some aspects of the questionnaire (e.g. perceptions of supervisors) will remind participants of upsetting experiences. There will be some inconvenience linked to the time taken to complete surveys. There is a potential risk related to loss of confidentiality but the information generated from this research will be kept confidential and the data collected will collect any personal identifying data.
